# *Rothia* from the Human Nose Inhibit *Moraxella catarrhalis* Colonization with a Secreted Peptidoglycan Endopeptidase

**DOI:** 10.1128/mbio.00464-23

**Published:** 2023-04-03

**Authors:** Reed M. Stubbendieck, Eishika Dissanayake, Peter M. Burnham, Susan E. Zelasko, Mia I. Temkin, Sydney S. Wisdorf, Rose F. Vrtis, James E. Gern, Cameron R. Currie

**Affiliations:** a Department of Bacteriology, University of Wisconsin-Madison, Madison, Wisconsin, USA; b Department of Microbiology and Molecular Genetics, Oklahoma State University, Stillwater, Oklahoma, USA; c Department of Pediatrics, University of Wisconsin School of Medicine and Public Health, Madison, Wisconsin, USA; d Microbiology Doctoral Training Program, University of Wisconsin-Madison, Madison, Wisconsin, USA; e Department of Medicine, University of Wisconsin School of Medicine and Public Health, Madison, Wisconsin, USA; f David Braley Centre for Antibiotic Discovery, Department of Biochemistry and Biomedical Sciences, McMaster University, Hamilton, Ontario, Canada; University of Connecticut

**Keywords:** asthma, acute otitis media, *Moraxella catarrhalis*, nasal microbiome, peptidoglycan endopeptidase, *Rothia aeria*, *Rothia dentocariosa*, *Rothia mucilaginosa*, *Rothia*, *asthma*

## Abstract

Moraxella catarrhalis is found almost exclusively within the human respiratory tract. This pathobiont is associated with ear infections and the development of respiratory illnesses, including allergies and asthma. Given the limited ecological distribution of M. catarrhalis, we hypothesized that we could leverage the nasal microbiomes of healthy children without M. catarrhalis to identify bacteria that may represent potential sources of therapeutics. *Rothia* was more abundant in the noses of healthy children compared to children with cold symptoms and M. catarrhalis. We cultured *Rothia* from nasal samples and determined that most isolates of Rothia dentocariosa and “Rothia similmucilaginosa” were able to fully inhibit the growth of M. catarrhalis *in vitro*, whereas isolates of Rothia aeria varied in their ability to inhibit M. catarrhalis. Using comparative genomics and proteomics, we identified a putative peptidoglycan hydrolase called secreted antigen A (SagA). This protein was present at higher relative abundance in the secreted proteomes of *R. dentocariosa* and *R. similmucilaginosa* than in those from non-inhibitory *R. aeria*, suggesting that it may be involved in M. catarrhalis inhibition. We produced SagA from *R. similmucilaginosa* in Escherichia coli and confirmed its ability to degrade M. catarrhalis peptidoglycan and inhibit its growth. We then demonstrated that *R. aeria* and *R. similmucilaginosa* reduced M. catarrhalis levels in an air-liquid interface culture model of the respiratory epithelium. Together, our results suggest that *Rothia* restricts M. catarrhalis colonization of the human respiratory tract *in vivo*.

## INTRODUCTION

The human nasal cavity is colonized by a low-diversity microbial community that includes commensals, mutualists, and opportunistic pathogens (also called “pathobionts”) (1–3). Nasal pathobionts include Staphylococcus aureus and Streptococcus pneumoniae (4), which can cause disease at body sites outside the nose (e.g., blood, heart, central nervous system, and skin) (5–8). The burden that S. aureus and S. pneumoniae represent on human health has been reviewed extensively (9–14). However, in addition to these well-characterized pathobionts, the nasal cavity is also home to other potential pathogens, including Moraxella catarrhalis. This Gram-negative pathobiont is found almost exclusively within the human respiratory tract and is an opportunistic pathogen in children and adults with chronic respiratory diseases.

Moraxella catarrhalis is an otopathogen and is increasingly isolated as a causative agent of acute otitis media (AOM; i.e., ear infection) in children (15). Between 2008 and 2014, the rate of AOM among children in the United States remained constant (16), despite the introduction of a 13-valent pneumococcal vaccine in 2008 that demonstrably reduced the burden of AOM caused by the major otopathogen S. pneumoniae (17). During the same period, there was a concomitant increase from 15% to 25% in the incidence of M. catarrhalis isolated from children with AOM (15, 18, 19). Moreover, over 85% of clinical isolates of M. catarrhalis produce chromosomally encoded β-lactamases, rendering them resistant to peptidoglycan-targeting β-lactam antibiotics, including first-line empirical agents used to treat AOM (i.e., amoxicillin) (20, 21). These β-lactamases can also protect other otopathogens, including nontypeable Haemophilus influenzae and S. pneumoniae, from antibiotics in mixed-species biofilms (22–24). Furthermore, M. catarrhalis colonization of the nasopharynx is correlated with the development of respiratory diseases, including allergies and asthma (25, 26). For instance, in a longitudinal birth cohort study, detection of *Moraxella* during incidents of wheezing illness in the first 3 years of life was strongly associated with the development of persistent asthma in adulthood (27). Furthermore, M. catarrhalis is increased in respiratory secretions during acute wheezing illnesses and contributes to exacerbations of asthma and chronic obstructive pulmonary disease (COPD) (25, 28, 29). Currently, no clinical trials of vaccines for M. catarrhalis for children have been conducted or are ongoing. In addition, vaccines against M. catarrhalis for adults with COPD have not been proven to be effective at preventing disease exacerbations or stimulating a persistent immune response against this pathobiont (30–33). Given this intrinsic antibiotic resistance, the lack of vaccines, and the economic burden of these diseases, it is of interest to identify alternative therapeutics that target M. catarrhalis.

The human nasal cavity is a high-stress, low-resource environment (34, 35). As one means of persisting in this hostile environment, bacteria engage in competitive interspecies interactions (recently reviewed by Hardy and Merrell [36]). The competitive mechanisms that bacteria use can be broadly categorized as exploitation or interference (37). In exploitation competition, one organism prevents its competitors from accessing resources, such as nutrients or space. For instance, Corynebacterium propinquum from the nasal cavity produces the siderophore dehydroxynocardamine and sequesters iron from coagulase-negative staphylococci to mediate exploitation competition (38). In contrast, interference competition involves the production of toxic effectors that directly kill or inhibit the growth of competitors. As examples in the nasal cavity, Staphylococcus lugdunensis produces the antibiotic lugdunin to inhibit S. aureus (39), several *Gammaproteobacteria* species produce antimicrobial peptides with activity against S. aureus (40), and Staphylococcus epidermidis produces the antibiotic nukacin IVK45 to inhibit various bacterial species, including M. catarrhalis (41). In addition to antibiotics, bacteria can also use enzymes and other secreted proteins to mediate inference competition. For example, in the nasal cavity, Corynebacterium accolens secretes a lipase that cleaves human triacylglycerides to release short-chain fatty acids with activity against S. pneumoniae (42). Because M. catarrhalis is specialized for the human respiratory tract, we hypothesized that other bacteria which colonize the nasal cavity may have evolved mechanisms to compete with this pathobiont in its native environment. These nasal bacteria could represent an avenue for the identification of therapeutics against M. catarrhalis.

In this study, we characterized the nasal bacterial communities of children with and without M. catarrhalis colonization. Among the bacterial taxa that were more abundant in the noses of healthy children without M. catarrhalis colonization than in children with cold symptoms and M. catarrhalis, we identified *Rothia* spp. (phylum *Actinobacteria*, family *Micrococcaceae*). We isolated *Rothia* from nasal lavage samples and determined that Rothia dentocariosa and a potentially novel species similar to Rothia mucilaginosa, which we provisionally named “*Rothia similmucilaginosa*,” were able to fully inhibit the growth of M. catarrhalis in *in vitro* coculture assays. In contrast, isolates of Rothia aeria were more variable in their ability to inhibit M. catarrhalis *in vitro*. Using a combination of comparative genomics and proteomics, we identified a putative peptidoglycan hydrolase called secreted antigen A (SagA). The SagA protein was present at higher relative abundance in the secreted proteomes of *R. dentocariosa* and *R. similmucilaginosa* than in those from *R. aeria*. To determine whether SagA can degrade M. catarrhalis peptidoglycan, we heterologously produced the protein and demonstrated its activity using zymography and against M. catarrhalis cultures. Finally, we showed that *R. aeria* and *R. similmucilaginosa* can protect cultures of differentiated airway epithelial cells from M. catarrhalis infection. Together, these data suggest that *Rothia* in the human nasal cavity may protect the host against M. catarrhalis colonization using the peptidoglycan endopeptidase SagA.

## RESULTS

### The nasal bacterial communities of healthy and children with cold symptoms and *Moraxella* colonization are distinct.

We were interested in identifying commensal nasal bacteria that can inhibit the growth of M. catarrhalis. As a first step to identify these candidates, we compared the communities of healthy children and children with cold symptoms and PCR-confirmed M. catarrhalis colonization from a subset of nasal lavage specimens collected as part of the Genetic Influences on Rhinovirus-Influenced Asthma (RhinoGen) study (25, 43) (see Table S1 in the supplemental material). We extracted total DNA from specimens collected from 10 children with varying degrees of cold symptoms (median age: 7.95 years, interquartile range [IQR]: 6.6 to 8.2 years; 100% male; median cold severity score: 7, IQR: 6 to 8) and 10 children with no symptoms (median age: 8.05 years, IQR: 8 to 8.2 years; 60% male; cold severity score: 0) and used 16S rRNA gene V4 region amplicon sequencing to characterize their nasal bacterial communities. In total, we identified 235 operational taxonomic units (OTUs) at ≥97% sequence identity from the 20 nasal communities (Table S2). There was a slight but significant difference in the Shannon diversity index between the nasal communities isolated from healthy children (mean = 1.88, standard deviation [SD] = 0.79) and those from children exhibiting any degree of cold symptoms (mean = 1.19, SD = 0.47; Wilcoxon rank-sum test: *P* = 0.029).

10.1128/mbio.00464-23.1 TABLE S1 Metadata and amplicon sequencing statistics for nasal lavage samples used in this study. Download Table S1, TXT file, 0.00 MB. Copyright © 2023 Stubbendieck et al. 2023 Stubbendieck et al. https://creativecommons.org/licenses/by/4.0/ This content is distributed under the terms of the Creative Commons Attribution 4.0 International license.

**FIG 1:**
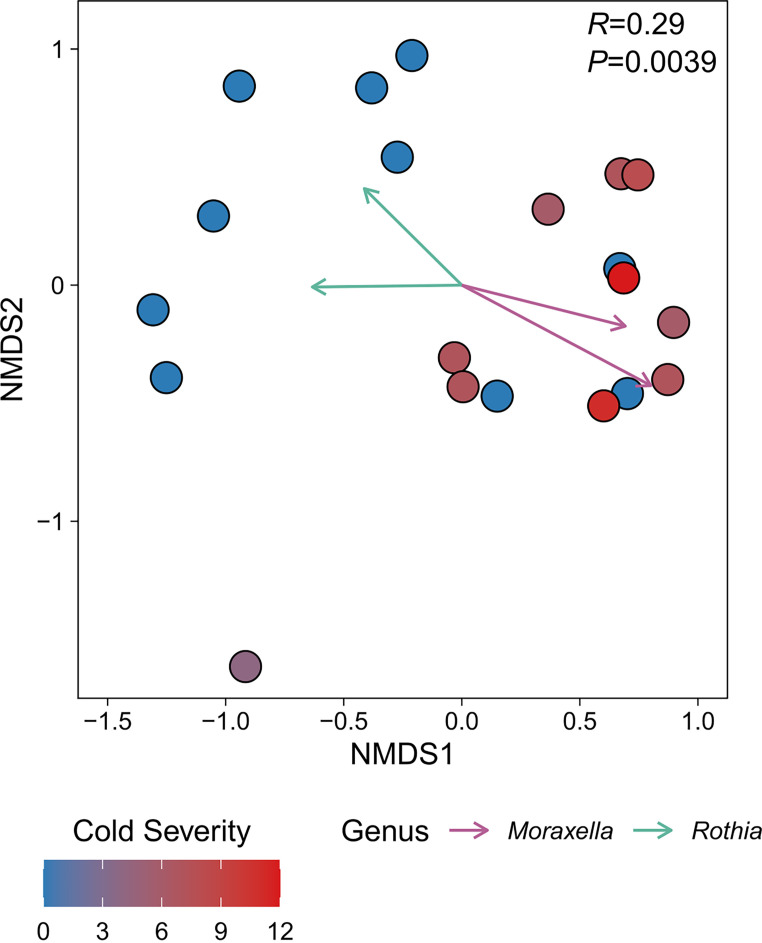
Bacterial community composition varies between children based on *Moraxella* colonization and correlates with cold severity. Each point in the nonmetric multidimensional scaling (NMDS) plot represents the bacterial community sequenced from one individual. Points are colored according to cold severity score (see Materials and Methods), as indicated by the key in the lower left. Vectors represent operational taxonomic units (OTUs) corresponding to either *Moraxella* or *Rothia.* Each vector originates from 0,0 and indicates a significant (α = 0.05) trend for select OTUs and their length is scaled according to the square root of their correlation coefficients such that longer vectors indicate stronger correlations. Vectors are colored by their genus-level identification, as indicated by the key in the lower right. The ANOSIM *R* and *P* values (100,000 permutations) for comparing children with upper respiratory tract infection symptoms (cold severity score > 0) with healthy children (cold severity score = 0) are shown in the top right of the plot. The stress value for the NMDS plot was 0.14.

10.1128/mbio.00464-23.2 TABLE S2 OTUs identified in 20 nasal communities analyzed in this study. The nonmetric multidimensional scaling (NMDS) coordinates, correlation coefficients (*R^2^*), and *P* values for fitting operational taxonomic unit (OTU) vectors across ordination space correspond to Fig. 1. Download Table S2, TXT file, 0.04 MB. Copyright © 2023 Stubbendieck et al. 2023 Stubbendieck et al. https://creativecommons.org/licenses/by/4.0/ This content is distributed under the terms of the Creative Commons Attribution 4.0 International license.

To determine whether nasal bacterial community composition was associated with donor health status, we investigated beta diversity using Bray-Curtis dissimilarity and nonmetric multidimensional scaling (NMDS). In general, we found that the bacterial communities from healthy children were distinct from those from children with cold symptoms (analysis of similarity [ANOSIM] *R *= 0.29, *P* = 0.0039) (Fig. 1). However, three nasal bacterial communities from healthy children grouped with nine of the communities from children with cold symptoms. In addition, one of the bacterial communities from a child with minor symptoms (cold severity score = 4) did not group with any other community in this analysis (Fig. 1). We identified 45 bacterial OTUs with significant trends (α = 0.05) across the ordination space (Table S2). Otu0001 (*R^2^* = 0.82, *P* = 0.00001) and Otu0222 (*R^2^* = 0.51, *P* = 0.0023), both corresponding to *Moraxella* spp., were strongly associated with communities isolated from children with cold symptoms. In contrast, we identified 24 OTUs that were associated with healthy communities. These OTUs included Otu0034 (*R^2^* = 0.40, *P* = 0.024) and Otu0039 (*R^2^* = 0.34, *P* = 0.047), which correspond to Rothia mucilaginosa (phylum *Actinobacteria*, family *Micrococcaceae*) and an unclassified *Rothia* sp., respectively.

### *Rothia* specifically inhibit *Moraxella* species.

Given the negative correlation of *Rothia* OTUs with *Moraxella* OTUs (Fig. 1), we hypothesized that these bacteria may be able to inhibit the growth of M. catarrhalis. To test this hypothesis, we first isolated *Rothia* from frozen nasal lavage specimens donated as part of the RhinoGen study. In addition, we supplemented these isolates with two strains that we previously isolated from specimens donated as part of the Childhood Origins of Asthma (COAST) study (38, 44, 45).

**FIG 2:**
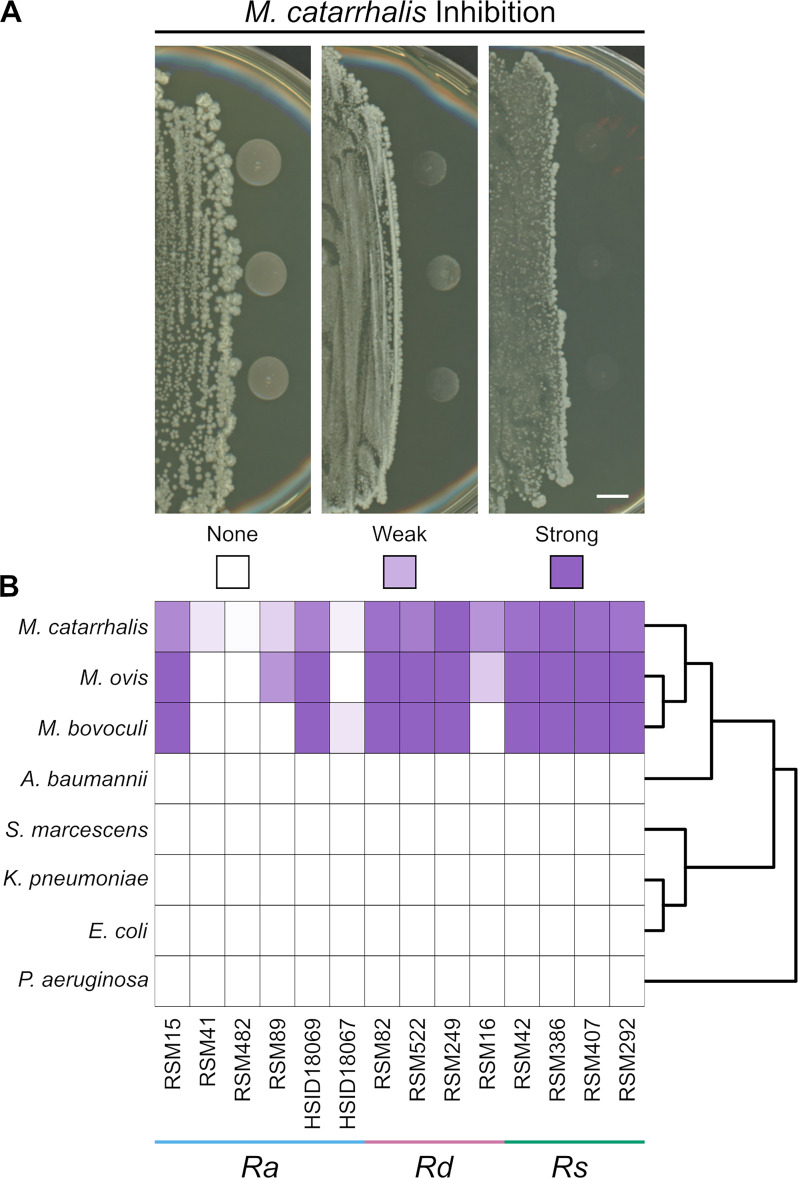
Inhibition of Moraxella catarrhalis by *Rothia*. (A) Cocultures of *Rothia* isolates (left side of image) and three colonies of M. catarrhalis strain O35E (right side of image) The inhibition score is indicated below each image, with the heat map key shown in panel B. Photographs were taken after co-incubation overnight on brain heart infusion (BHI) agar and are representative of ≥3 replicates. For M. catarrhalis, the inhibition score represents the aggregate for six different strains. See Fig. S1 for the results of each M. catarrhalis strain individually. Scale bar = 5 mm. (B) Heat map shows the inhibition scores of each *Gammaproteobacteria* strain (left) when paired with the corresponding *Rothia* isolate (below). Each interaction was replicated ≥3 times and the inhibition scores were averaged. Right side shows cladogram of the *Gammaproteobacteria* strains used in this study. Abbreviations: *Ra*, *R. aeria*; *Rd*, *R. dentocariosa*; *Rs*, *R. similmucilaginosa*.

We assessed the ability of a subset of the cultured *Rothia* isolates to inhibit the growth of M. catarrhalis *in vitro* using pairwise inhibition assays. We cultured 14 different *Rothia* isolates on brain heart infusion (BHI) agar plates. After the colonies were established, we inoculated six different M. catarrhalis strains next to the *Rothia* colony, then scored them for inhibition by visual assessment of the coculture after overnight incubation. Consistent with our hypothesis, we found that some *Rothia* isolates were able to inhibit the growth of multiple M. catarrhalis strains. In general, we did not observe differences in inhibition among different M. catarrhalis strains (Fig. S1). Across technical replicates of our assays, we generally observed concordance in M. catarrhalis inhibition. However, between different experimental replicates, *Rothia* isolates that generally inhibited M. catarrhalis or had no effect on its growth sometimes showed weak inhibition in another replication. Therefore, we assigned each *Rothia* isolate into one of three categories based on its average ability to inhibit M. catarrhalis: strong inhibition (fully inhibited most M. catarrhalis strains in nearly all replicates, *n* = 7), weak inhibition (partially inhibited most M. catarrhalis strains in nearly all replicates or had variable inhibition across replicates, *n* = 3), and no inhibition (did not inhibit most M. catarrhalis strains in nearly all replicates, *n* = 4) (Fig. 2A).

10.1128/mbio.00464-23.4 FIG S1 Strains of Moraxella catarrhalis are inhibited by *Rothia*. Heat map displays the inhibition scores of each M. catarrhalis strain (left) when paired with the corresponding *Rothia* isolate (below). Each interaction was replicated ≥3 times and the inhibition scores were averaged. Abbreviations: *Ra*, *R. aeria*; *Rd*, *R. dentocariosa*; *Rs*, *R. similmucilaginosa*. Download FIG S1, TIF file, 0.3 MB. Copyright © 2023 Stubbendieck et al. 2023 Stubbendieck et al. https://creativecommons.org/licenses/by/4.0/ This content is distributed under the terms of the Creative Commons Attribution 4.0 International license.

We then wanted to determine whether this inhibition was specific to M. catarrhalis or whether other *Gammaproteobacteria* were susceptible to inhibition by the *Rothia* isolates. We repeated the inhibition assays and included strains of Acinetobacter baumannii, Escherichia coli, Klebsiella pneumoniae, Pseudomonas aeruginosa, and Serratia marcescens. In addition, we tested whether these *Rothia* isolates inhibited the growth of Moraxella bovoculi and Moraxella ovis, which were isolated from a cow and sheep, respectively, with conjunctivitis (Fig. 2B). In general, we observed that all three *Moraxella* species behaved identically in the inhibition assays (i.e., if a *Rothia* isolate inhibited M. catarrhalis, then it also inhibited *M. bovoculi* and *M. ovis*). We never observed inhibition of a non-*Moraxella* species by any of the 14 *Rothia* isolates that we tested (Fig. 2B). These 14 *Rothia* isolates were also tested for activity against a panel containing three fungi (Aspergillus flavus, Candida albicans, and Trichosporon asahii), three Gram-positive bacteria (Bacillus subtilis, Enterococcus faecalis, and S. aureus), and one additional Gram-negative bacterium (Enterobacter cloacae). No inhibitory activity was observed against any fungi or bacteria, except *Moraxella* species. Taken together, these data indicate that some strains of *Rothia* produce factor(s) that specifically inhibit *Moraxella* at a distance.

### Different species of *Rothia* are associated with inhibition and non-inhibition of *M. catarrhalis*.

**FIG 3:**
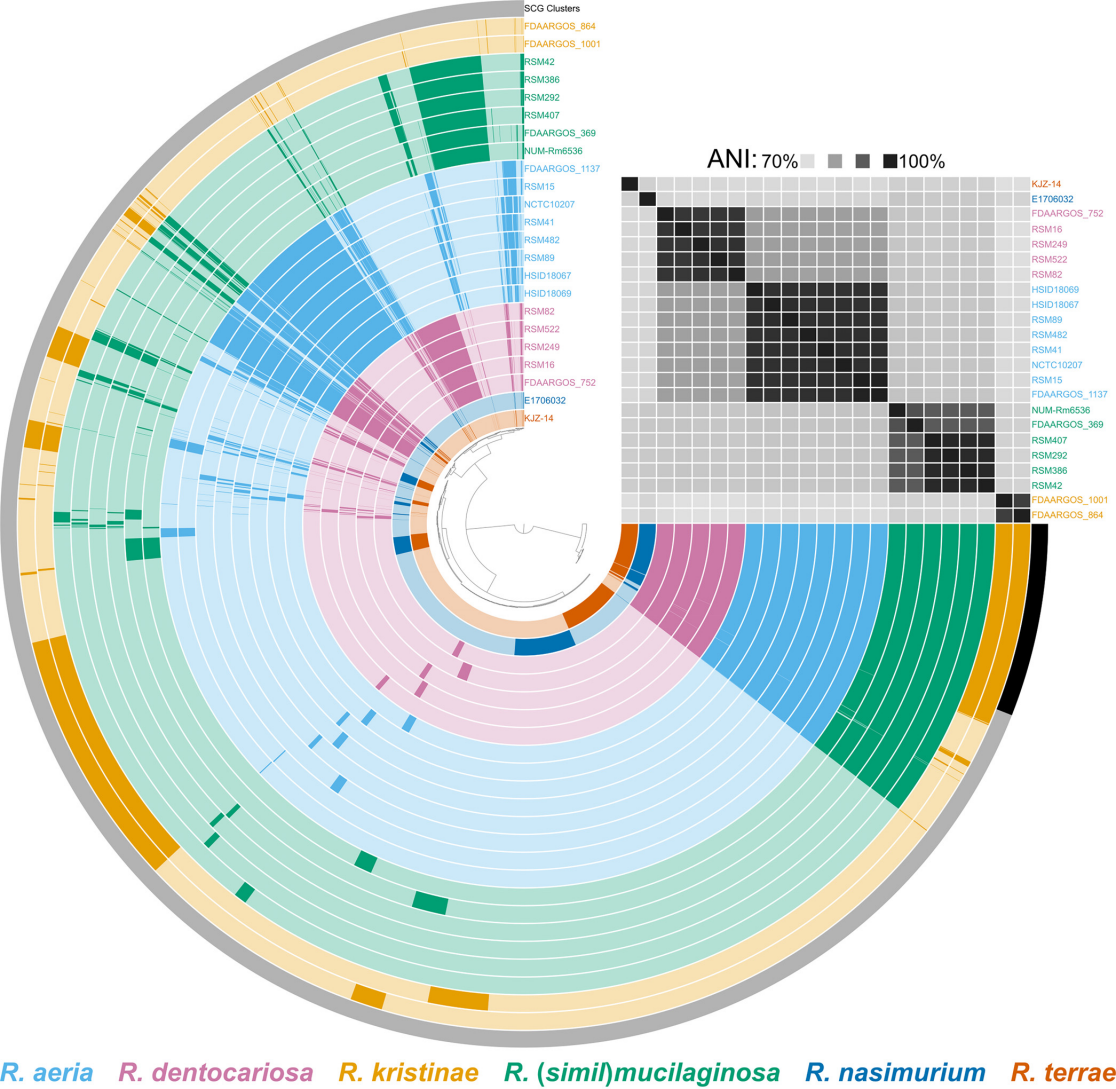
Pangenome of *Rothia*. This pangenome was generated from 9 reference genomes and 14 draft genome sequences of the strains used in this study using the anvi’o 7 pangenome pipeline. Each arc represents a single genome and is colored based on its species-level identification, as indicated by the key below. Genomes are ordered based on average nucleotide identity (ANI; top right). In total, this pangenome contains 48,332 genes within 8,099 gene clusters. The presence or absence of a gene cluster within a given genome is indicated by opaque and transparent colors, respectively. Black bar indicates the set of single-copy core gene (SCG) clusters.

To confirm the identity of each *Rothia* isolate, we extracted and sequenced genomic DNA to generate draft genome sequences for each isolate used in the coculture assays. We then compared these genome sequences with 9 complete, high-quality *Rothia* genomes from GenBank, including *R. aeria*, *R. dentocariosa*, *R. kristinae*, *R. mucilaginosa*, *R. nasimurium*, and *R. terrae*. In addition, we constructed a core-genome phylogeny from 93 conserved single-copy genes using these same genome sequences. Based on gene content, average nucleotide identity (ANI), and phylogenetic placement (Fig. 3, Fig. S2), we identified the 14 *Rothia* isolates used in this study as *R. aeria* (*n* = 6, ANI = 97.7% to 99.5% relative to reference strain FDAARGOS_1137), *R. dentocariosa* (*n* = 4, ANI = 96.5% to 96.8% relative to reference strain FDAARGOS_752), and a species closely related to *R. mucilaginosa* (*n* = 4, ANI: 91.5% to 91.6% relative to reference strain FDAARGOS_369). We note that though the isolates in the latter category were closely related to *R. mucilaginosa*, they did not reach the frequently used 95% ANI cutoff for species delineation (46). In addition, these four isolates did not reach this ANI threshold compared with any other characterized *Rothia* stain. Henceforth, for simplicity, we will refer to these isolates using the provisional species name “*Rothia similmucilaginosa*.” A full biochemical characterization and formal classification of these *R. similmucilaginosa* isolates is forthcoming. We found that three *R. dentocariosa* isolates and all four *R. similmucilaginosa* isolates strongly inhibited M. catarrhalis. Two *R. aeria* isolates and one *R. dentocariosa* isolate weakly inhibited M. catarrhalis. The remaining four *R. aeria* isolates did not inhibit M. catarrhalis (Fig. 2B). We next sought to determine the identity of the anti-*Moraxella* inhibitory factor(s) produced by the inhibitory *Rothia* isolates.

10.1128/mbio.00464-23.5 FIG S2 Core-genome phylogeny of *Rothia*. The phylogenetic tree was built from 9 reference strains of *R. aeria*, *R. dentocariosa*, *R. kristinae*, *R. mucilaginosa*, *R. nasimurium*, and *R. terrae* and 14 *Rothia* isolates used in this study. The *Rothia* strains used for inhibition assays are in bold. The phylogeny is rooted on Kocuria rosea ATCC 186 (phylum *Actinobacteria*, order *Actinomycetales*, family *Micrococcaceae*) and branch lengths were transformed to be proportional to the root. Nodes with ≥75% bootstrap support (100 replicates) are labeled with the corresponding percentage. Download FIG S2, TIF file, 0.3 MB. Copyright © 2023 Stubbendieck et al. 2023 Stubbendieck et al. https://creativecommons.org/licenses/by/4.0/ This content is distributed under the terms of the Creative Commons Attribution 4.0 International license.

### Inhibition of *M. catarrhalis* by *Rothia* is not due to production of a siderophore.

*Rothia* genomes contain biosynthetic gene clusters (BGCs) for the production of antibiotics and other secondary metabolites (47–49). We hypothesized that the *Rothia* strains with inhibitory activity against M. catarrhalis produce secondary metabolite(s) that the non-inhibitory strains lack. To test this hypothesis, we first identified BGCs contained within *Rothia* genomes using antiSMASH (50) (Fig. S3A). Consistent with a previous study that investigated the biosynthetic capacity of *Rothia* genomes (51), we identified a single BGC encoding the production of the non-ribosomal peptide siderophore enterobactin that was present in the genomes of all 14 isolates used in this study and 8 of the reference genomes (Fig. S2A). For all *R. similmucilaginosa* genomes, the enterobactin BGC was the only BGC we detected (Fig. S3A).

10.1128/mbio.00464-23.6 FIG S3 Inhibition of M. catarrhalis by *Rothia* is not due to siderophore production. (A) Heat map of secondary metabolite biosynthetic gene clusters (BGCs; columns) predicted in *Rothia* genomes (rows). The presence or absence of a BGC in each genome is indicated by black or white, respectively. *Rothia* strains are colored according to the species, as indicated by the key below the heat map. No BGCs were detected in *R. nasimurium* strain E1706032. (B) CAS assay results from *R. aeria* strain RSM41 (left), *R. dentocariosa* strain RSM522 (middle), and *R. similmucilaginosa* strain RSM42 (right). A shift in the color of the chrome azurol S (CAS) overlay from blue to yellow indicates siderophore production. Note: color appears greenish when overlaid on brain heart infusion (BHI) medium, which is yellow in color. (C) Cocultures of the same *Rothia* isolates from panel B (left side of image) and three colonies of M. catarrhalis strain O35E (right side of image) on conventional BHI medium (left) and BHI medium supplemented with 200 μM FeCl_3_ (+FeCl_3_) (right). Photographs were taken after co-incubation overnight on BHI agar and are representative of 3 replicates. Scale bars in panels B and C are 5 mm. Download FIG S3, TIF file, 4.9 MB. Copyright © 2023 Stubbendieck et al. 2023 Stubbendieck et al. https://creativecommons.org/licenses/by/4.0/ This content is distributed under the terms of the Creative Commons Attribution 4.0 International license.

Siderophores are molecules produced by microbes to scavenge ferric iron and other minerals from the environment (52). In a previous study, we determined that *R. aeria* strains HSID18067 and HSID18069 did not produce siderophore activity under similar, but slightly different, assay conditions to those used in the present study (38). Here, these strains possessed no activity and partial activity against M. catarrhalis, respectively (Fig. 2B). Therefore, we wanted to determine if *R. dentocariosa* and *R. similmucilaginosa* strains produce siderophores as a potential mechanism to inhibit M. catarrhalis growth. Using chrome azurol S (CAS) assays, we detected no iron sequestration from *R. aeria* strain RSM41, *R. dentocariosa* strain RSM522, or *R. similmucilaginosa* strain RSM42 under the same conditions as used for the coculture inhibition assays (Fig. S3B), suggesting that these bacteria do not produce siderophores. In a parallel approach to test whether iron sequestration by *Rothia* is responsible for inhibition of M. catarrhalis, we performed inhibition assays using standard BHI agar plates ±200 μM ferric chloride (FeCl_3_). We found that iron supplementation did not appreciably change the inhibition phenotype of M. catarrhalis strain O35E when cultured with *R. similmucilaginosa* strain RSM42 or *R. dentocariosa* strain RSM522 (Fig. S3C). Taken together, these data indicate that iron limitation via a siderophore or production of another metabolite by *R. dentocariosa* and *R. similmucilaginosa* are not responsible for the observed inhibition of M. catarrhalis.

### Identification of secreted proteins associated with *Rothia* strains that inhibit *M. catarrhalis*.

Given the previous finding that M. catarrhalis inhibition was likely not due to production of enterobactin or another secondary metabolite (Fig. S3), we hypothesized that *Rothia* may produce a protein that inhibits this pathobiont.

**FIG 4:**
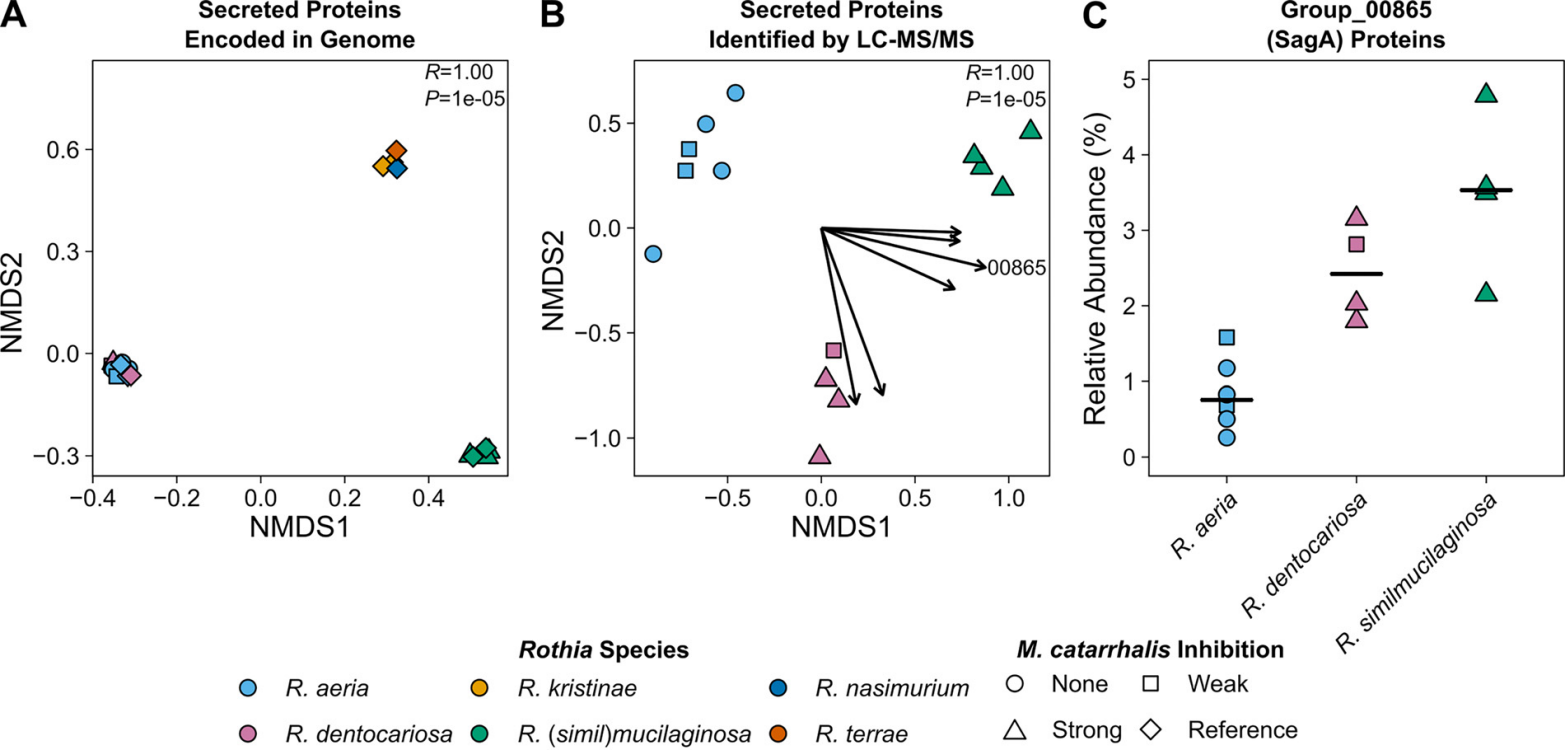
The secreted proteomes of *Rothia* vary by species. (A) Each point in the NMDS plot represents the presence or absence of 448 groups of homologous proteins with predicted secretion signals within a single *Rothia* genome. Points have been jittered to avoid overplotting. The ANOSIM *R* and *P* values (100,000 permutations) for comparing species are shown in the top right of the plot. The stress value for the NMDS plot was 1e–04. (B) Proteins secreted into agar by *Rothia* were extracted and identified using untargeted liquid chromatography-tandem mass spectrometry (LC-MS/MS). Each point in the NMDS plot represents the relative abundance of 181 groups of homologous proteins with predicted secretion signals detected within a single proteome. Each vector originates from 0,0 and indicates a significant (α = 0.05) trend for a subset of homologous proteins (Table 1), and their length is scaled according to the square root of their correlation coefficients such that longer vectors indicate stronger correlations. The vector for group_00865 (SagA) is labeled. The ANOSIM *R* and *P* values (100,000 permutations) for comparing species are shown in the top right of the plot. The stress value of the NMDS plot was 0.084. (C) The relative abundance of homolog group_00865, which corresponds to SagA (secreted antigen A), in the secreted proteomes of *Rothia* grouped by species. Horizontal black bars indicate the median relative abundance values. The points in all plots are colored based on the species-level identification and the shapes correspond to the M. catarrhalis inhibition phenotype, as indicated by the key below the plots.

We compared the genomes of different *Rothia* to determine whether the inhibitory strains encode proteins that were absent in the genomes of the non-inhibitory strains. When we analyzed the pangenome of *Rothia*, we determined that there was a strong signal between overall gene content and *Rothia* species-level identity (Fig. 3). Because inhibition of M. catarrhalis by *Rothia* occurred at a distance, we reasoned that an inhibitory protein must be secreted. Therefore, we decided to focus our subsequent analysis on genes that encode proteins with identifiable secretion signals. Of the 4,086 total groups of homologous protein-encoding genes we identified in the *Rothia* pangenomes, 488 (12% total) encoded proteins with predicted secretion signals. We observed three clusters of *Rothia* based on the presence or absence of homologous groups of genes encoding secreted proteins. The first cluster consisted of *R. aeria* and *R. dentocariosa*, the second cluster consisted of *R. kristinae*, *R. nasimurium*, and *R. terrae*, and the final cluster consisted of *R. mucilaginosa* and *R. similmucilaginosa* (Fig. 4A). These clusters are broadly consistent with *Rothia* phylogeny (Fig. S2) but did not inform the identification of a protein secreted by both *R. dentocariosa* and *R. similmucilaginosa* that inhibits M. catarrhalis. In addition, we did not identify any homologous groups of genes encoding secreted proteins that were universally shared between *R. dentocariosa* and *R.* (*simil*)*mucilaginosa* but were absent in *R. aeria*. Further, given that *R. aeria* strains HSID18069 and RSM15 had weak activity against M. catarrhalis (Fig. 2B), it is possible that these strains also produce inhibitory protein(s), albeit at lower levels.

To narrow the list of candidates for the identity of M. catarrhalis inhibitory protein(s), we characterized the secreted proteomes of the *Rothia* isolates that we used for inhibition assays. We cultured *R. aeria*, *R. dentocariosa*, and *R. similmucilaginosa* as before, but excised the agar near the bacterial colonies where we would inoculate M. catarrhalis in a coculture inhibition assay and extracted proteins. We then used untargeted liquid chromatography-tandem mass spectrometry (LC-MS/MS) to identify proteins secreted by each *Rothia* isolate. In total, we identified 181 homologous groups of secreted proteins from the three *Rothia* species. In contrast to the clustering observed when considering the content of genes encoding secreted proteins (Fig. 4A), we found that *R. aeria*, *R. dentocariosa*, and *R. similmucilaginosa* each formed their own species-based clusters (ANOSIM *R *= 1.00, *P* = 0.00001) (Fig. 4B). When we searched the secreted proteomes for proteins that were associated with both *R. dentocariosa* and *R. similmucilaginosa*, we identified six groups of homologous secreted proteins (Fig. 4B, Table 1). The proteins represented by homolog group_00865 emerged as candidates of interest for the anti-M. catarrhalis activity. These proteins were found in higher relative abundance in the secreted proteomes of *R. dentocariosa* (median: 2.4% of proteins with secretion signal) and *R. similmucilaginosa* (median: 3.5% of proteins with secretion signal) than in that of *R. aeria* (median: 0.75% of proteins with secretion signal) (Fig. 4C). In the *R. mucilaginosa* strain Num-Rm6536 reference genome, the gene encoding the group_00865 protein homolog is named *secreted antigen A* (*sagA*) (53). Therefore, we subsequently refer to this group of proteins as SagA proteins.

**TABLE 1:** Homologous protein groups associated with *R. dentocariosa* and *R. similmucilaginosa*^*a*^

Homolog group	Locus (name)^*b*^	*R^2^*	*P*	Predicted function^*c*^
00865	RM6536_0976 (SagA)	0.80	0.00001	C40 family peptidase homolog
01715	RM6536_1432 (hypothetical protein)	0.74	0.00059	Plasmid partitioning protein
01164	RM6536_1002 (extracellular protease precursor)	0.74	0.0013	S8 family peptidase
01704	RM6536_0570 (hypothetical protein)	0.59	0.012	ABC transporter family substrate-binding protein
00768	RM6536_1659 (FkpA)	0.55	0.012	FKBP-type peptidyl-prolyl *cis-trans* isomerase
01276	RM6536_1518 (dihydrolipoamide acetyltransferase component)	0.55	0.013	Alpha-mannosidase

a The *R^2^* and *P* values were obtained using the envfit function from the vegan package in R. SagA, secreted antigen A.

b Loci and names are based on the reference genome for *R. mucilaginosa* strain NUM-Rm6536. Product names are listed if known.

c Predicted function was determined by BLAST search of the homolog group consensus sequence against the nonredundant protein sequences database.

SagA proteins from *Rothia* spp. are ~20 kDa (197 to 210 amino acids), containing a 30-amino acid N-terminal signal peptide and a C-terminal endopeptidase, NlpC/P60 cysteine peptidase domain (InterPro accession no. IPR000064), and are homologous to members of the C40 peptidase family. Proteins with the NlpC/P60 domain have several known activities, including cleaving d*-*γ-glutamyl-meso-diaminopimelate or *N*-acetylmuramate-l-alanine linkages in peptidoglycan cross-bridges (i.e., these proteins possess peptidoglycan endopeptidase activity) (54). Based on alignment of SagA from *R. aeria* strain RSM41, *R. dentocariosa* strain RSM522, and *R. similmucilaginosa* strain RSM42 to each other and to structurally characterized C40 family peptidases, including the family type protein (MEROPS accession no. MER0001322) (55), we identified the conserved cysteine and histidine residues involved in the cysteine peptidase reaction mechanism (Fig. S4A). However, SagA is classified as a non-peptidase homolog in the MEROPS database because it has a serine (Ser-172), instead of a charged residue in the third position of the catalytic triad (Cys-His-[Asn/Gln/Glu/His]) (Fig. S4A). The charged residue does not directly participate in the reaction mechanism but is important for coordinating the basic histidine residue (corresponding to His-160 in SagA from *R. similmucilaginosa* strain RSM42). It is known that the eukaryotic endothelial protease vasohibin uses a Cys-His-Ser catalytic triad in which the serine residue orients the histidine base (56). We modeled 176 residues of SagA from *R. similmucilaginosa* strain RSM42 onto the C40 family peptidase RipA from Mycobacterium tuberculosis (Protein Data Bank [PDB] ID: 2XIV). Based on the homology model, we found that Ser-172 is positioned well to orient His-160 using a hydrogen bond (Fig. S4B). Therefore, we hypothesized that SagA is a functional peptidoglycan endopeptidase and may be responsible for inhibition of M. catarrhalis by *Rothia*.

10.1128/mbio.00464-23.7 FIG S4 Secreted antigen A (SagA) is a C40 family peptidase homologue. (A) Amino acid sequence alignment of SagA proteins from *Rothia* with structurally characterized C40 family peptidase homologues. Amino acids are colored based on side-chain chemistry. Residues in gray are identical to the reference sequence. The tick marks indicate the catalytic triad. The numbering on top and bottom of the alignment represents the ungapped positions in the reference MER0001322 sequence and *R. similmucilaginosa* strain RSM42 sequence, respectively. Underneath the positions on the bottom are the amino acids found in the canonical catalytic triad for C40 family peptidases. (B) Predicted structure of SagA from *R. similmucilaginosa* strain RSM42 using the structure of “Rv1477, hypothetical invasion protein of M. tuberculosis” (PDB ID: 2XIV) as the template. The putative residues in the catalytic triad are highlighted. The nitrogen, oxygen, and sulfur atoms are colored blue, red, and yellow, respectively. Abbreviations: Bs, Bacillus subtilis; Ec, Escherichia coli; Ls, Lysinibacillus sphaericus; Mt, Mycobacterium tuberculosis; Np, Nostoc punctiforme; Ra, Rothia aeria; Rd, Rothia dentocariosa; *Rs*, *Rothia similmucilaginosa*. Download FIG S4, TIF file, 1.4 MB. Copyright © 2023 Stubbendieck et al. 2023 Stubbendieck et al. https://creativecommons.org/licenses/by/4.0/ This content is distributed under the terms of the Creative Commons Attribution 4.0 International license.

### SagA degrades peptidoglycan from *M. catarrhalis* and inhibits its growth.

Currently, genetic manipulation of *Rothia* is challenging and the necessary conditions for transformation are not well-defined. Therefore, to further characterize SagA and determine its activity, we chose to use heterologous expression in E. coli.

We initially cloned a sequence encoding the C-terminal NlpC/P60 cysteine peptidase domain of SagA, without the N-terminal signal peptide, from *R. similmucilaginosa* strain RSM42 with an added Shine-Dalgarno sequence and start codon into an isopropyl β-d-1-thiogalactopyranoside (IPTG)-inducible expression vector. For simplicity, we subsequently refer to the truncated protein as SagA^37-197^. When we sequenced three different E. coli clones, we identified two different 32-bp deletions of the *lac* operator sequence. Given the propensity for IPTG-inducible systems to have leaky expression (57), we hypothesized that production of SagA^37-197^ in E. coli was cytotoxic and the deletions in the *lac* operator sequence prevented expression. Therefore, we chose to use an l-arabinose-inducible expression system, as these systems are more tightly controlled than IPTG-inducible systems (57). We confirmed dose-dependent production of the SagA^37-197^ fusion protein from the l-arabinose-inducible expression system by E. coli as an ~17.2-kDa protein using sodium dodecyl sulfate polyacrylamide gel electrophoresis (SDS-PAGE) (Fig. S5A). During these experiments, we noticed that E. coli producing SagA^37-197^ tended to grow 2 to 3× more slowly and formed smaller colonies when cultured on media containing l-arabinose, compared to uninduced controls (Fig. S5B). Taken together with the deletions observed in IPTG-inducible constructs, these data support the hypothesis that production of SagA^37-197^ is cytotoxic to E. coli, consistent with the activity of a peptidoglycan endopeptidase.

10.1128/mbio.00464-23.8 FIG S5 SagA production is cytotoxic to E. coli. (A) A strain of E. coli harboring a plasmid with an l-arabinose-inducible system for production of the SagA^37-197^ protein was cultured in LB supplemented with 0.002, 0.02, 0.2, and 0.2% (wt/vol) l-arabinose (A) for 3 h and harvested by centrifugation. Cultures were normalized by OD_600_ and whole-cell lysates were separated by SDS-PAGE. Negative controls for SagA^37-197^ production, uninduced (LB), and 2% (wt/vol) d-glucose repressed cultures were processed identically. Proteins were visualized using a Coomassie blue stain. Lane M shows molecular weight markers in kDa. The SagA^37-197^ protein band is indicated with an asterisk (*). Gel is representative of duplicate experiments. (B) The E. coli strain from panel A was cultured in LB with no supplementation, serially diluted, and spotted onto LB, LB containing 2% (wt/vol) d-glucose, and LB containing 2% (wt/vol) l-arabinose. Scale bar = 5 mm. Download FIG S5, TIF file, 0.5 MB. Copyright © 2023 Stubbendieck et al. 2023 Stubbendieck et al. https://creativecommons.org/licenses/by/4.0/ This content is distributed under the terms of the Creative Commons Attribution 4.0 International license.

**FIG 5:**
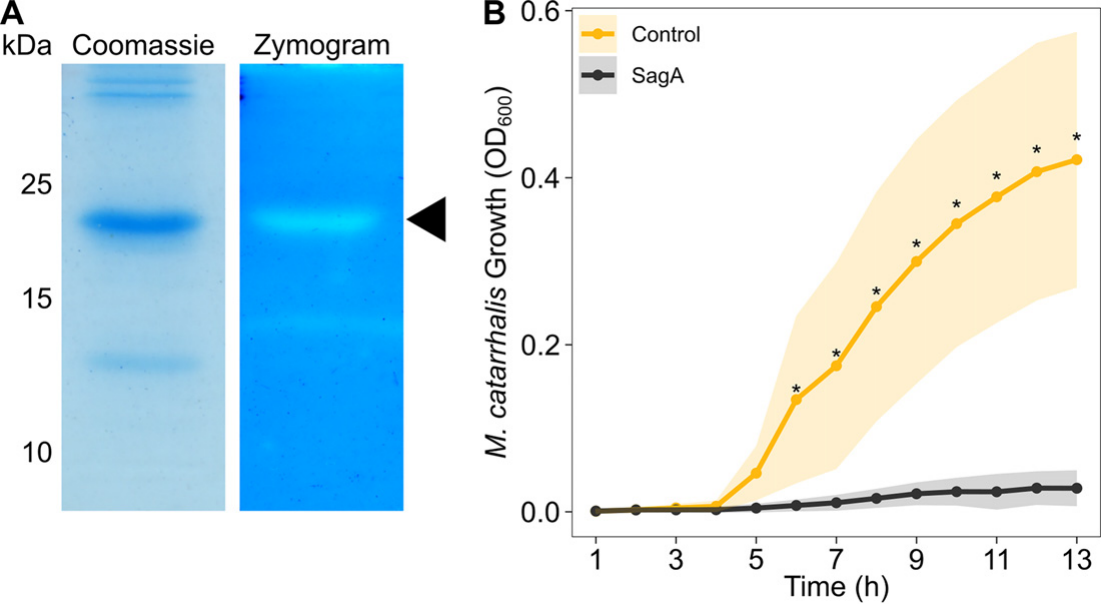
SagA inhibits M. catarrhalis through peptidoglycan degradation. (A) Two μg of partially purified 6×His-SagA^37-197^ was loaded onto a standard polyacrylamide gel stained with Coomassie brilliant blue G-250 (left) or onto a zymogram gel stained with methylene blue (right). For the zymogram gel, peptidoglycan from M. catarrhalis strain O35E was incorporated during polymerization. Peptidoglycan degradation is indicated by clearing in the zymogram gel. Molecular weight markers in kDa are shown to the left. The 6×His-SagA^37-197^ protein band is indicated with a triangle. Gels are representative of triplicate experiments. Note, the gel images were scaled to the same size. (B) Growth curve of M. catarrhalis strain O35E cultured for 13 h in the presence of 6×His-SagA^37-197^ (black) or an equivalent extract purified from E. coli harboring an empty pBAD30 plasmid (gold). Each inhibition assay was repeated with four separate preparations and two technical replicates. Points represent the average optical density at 600 nm (OD_600_) at each time point and shading indicates standard error. Asterisks (*) indicate time points at which a Wilcoxon rank-sum test demonstrated a significant difference (α = 0.05) in growth between the control and the culture exposed to 6×His-SagA^37-197^.

For subsequent experiments, we chose to optimize the coding sequence for SagA for expression in E. coli and added a 6×His tag for purification. We partially purified SagA from E. coli using nickel affinity chromatography and tested the protein for peptidoglycan degradation activity using zymography. We incorporated M. catarrhalis strain O35E peptidoglycan into a polyacrylamide gel during polymerization. Following electrophoresis, we renatured the proteins within the zymography gel, allowed them to hydrolyze M. catarrhalis peptidoglycan, and stained the remaining peptidoglycan using methylene blue. We confirmed that SagA degrades M. catarrhalis peptidoglycan, as indicated by a zone of clearing within the zymogram gel, corresponding to the same ~19.8-kDa band in the Coomassie-stained gel (Fig. 5A). Using LC-MS/MS, we confirmed that both of these bands corresponded to 6×His-SagA^37-197^.

We then wanted to determine if SagA directly inhibits M. catarrhalis growth. We cultured M. catarrhalis strain O35E in the presence of 6×His-SagA^37-197^ and assessed its growth over time. We found that M. catarrhalis cultures exposed to SagA failed to grow, whereas cultures exposed to an equivalent purification from E. coli cells harboring an empty vector were not inhibited (Fig. 5B).

### *Rothia* protect against *M. catarrhalis* colonization in cultures of respiratory epithelium.

To determine if *Rothia* species were protective against M. catarrhalis in models that are more representative of their natural environments, we chose to perform coculture assays on air-liquid interface (ALI) cultures of respiratory epithelium. We differentiated residual tissue from donor lungs into epithelial respiratory cells and colonized these cells with *R. aeria* strain RSM41, *R. dentocariosa* strain RSM522, or *R. similmucilaginosa* strain RSM42. Subsequently, we infected these cultures with M. catarrhalis strain O35E. After 24 h of coculture, we washed the apical surface, collected the epithelium, *Rothia*, and M. catarrhalis cells together, lysed the cells, and assessed M. catarrhalis colonization levels using a quantitative PCR (qPCR) assay. We report our results in CFU equivalents (CFUe), based on calibration of the qPCR cycle threshold with known quantities of M. catarrhalis in pure culture. As a control, we included a treatment where the epithelial cells were not colonized with *Rothia*.

**FIG 6:**
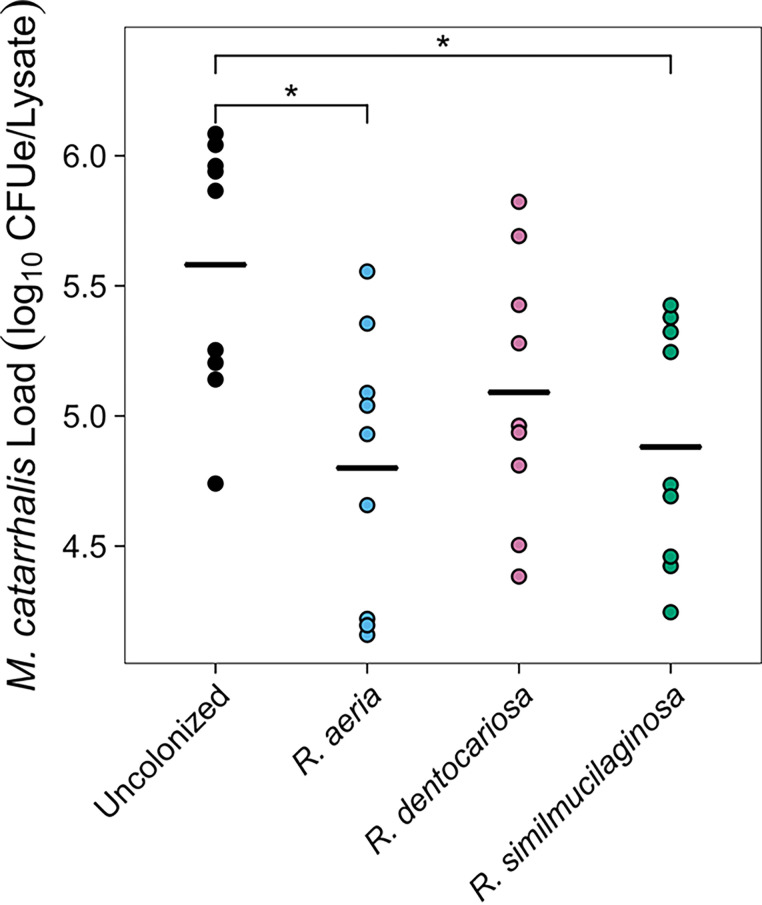
*Rothia* inhibit M. catarrhalis in respiratory epithelial cell culture. The apical surfaces of respiratory epithelial cell cultures were first colonized with *R. aeria* strain RSM41, *R. dentocariosa* strain RSM522, or *R. similmucilaginosa* strain RSM42 before subsequent infection with M. catarrhalis O35E. After 1 day of infection, the epithelial cells were collected and lysed, and the CFU equivalents (CFUe) of M. catarrhalis in each sample were quantified using quantitative PCR. Each point represents a result from a separate experiment. Horizontal black bars represent the mean abundance of M. catarrhalis for each treatment. Asterisks (*) indicate comparisons with significant difference (α = 0.05) after performing Tukey’s honestly significant difference (HSD) tests.

We performed cocultures of *Rothia* and M. catarrhalis on cell lines from two different donors. There was no significant effect of cell line (analysis of variance [ANOVA] *F*[1,32] = 0.53, *P* = 0.47) or interaction between cell line and *Rothia* species (ANOVA *F*[3,32] = 2.55, *P* = 0.073) in our linear model. Therefore, we chose to consider both cell lines together for subsequent analyses. We found that there was a significant effect of *Rothia* treatment on M. catarrhalis strain O35E levels (ANOVA *F*[3,36] = 2.94, *P* = 0.046). However, *post hoc* comparisons did not initially reveal significant differences between any of the treatments. This non-significance was driven by a single outlying experiment in which M. catarrhalis strain O35E failed to colonize the cells (1.4 × 10^4^ CFUe detected [est. <0.05% of the inoculum], compared to a median of 4.6 × 10^5^ CFUe [est. 1.5% of the inoculum]). Consequently, we repeated our analyses with this set of experiments excluded, including the matched *Rothia*-colonized ALI cultures. As expected, we found that there was a stronger effect of *Rothia* treatment on M. catarrhalis strain O35E levels (ANOVA *F*[3,32] = 4.50, *P* = 0.0096). Post hoc comparisons revealed that colonization of respiratory epithelial cells with either *R. aeria* strain RSM41 (Tukey’s honestly significant difference [HSD] *Q *= 4.72, adjusted *P* = 0.011) or *R. similmucilaginosa* strain RSM42 (Tukey’s HSD *Q *= 4.24, adjusted *P* = 0.026) decreased M. catarrhalis strain O35E colonization by ~0.8 log (Fig. 6). Unexpectedly, colonization of the epithelial cells with *R. dentocariosa* strain RSM522 did not result in a decrease in M. catarrhalis strain O35E levels (Fig. 6).

## DISCUSSION

In this study, we identified specific nasal bacteria that inhibit the growth of the pathobiont M. catarrhalis. We initially compared the nasal cavity bacterial communities of children with and without *Moraxella* colonization, which led us to determine that *Rothia* was associated with healthy nasal microbiomes that were free of M. catarrhalis colonization (Fig. 1). We performed targeted isolation of *Rothia* from nasal lavage samples. We then determined that *R. dentocariosa* and *R. similmucilaginosa* strains inhibited the growth of M. catarrhalis *in vitro*, while *R. aeria* strains tended to be less inhibitory (Fig. 2 and 3). We reasoned that the inhibitory factor produced by these *Rothia* spp. must be a secreted protein, so we characterized the secreted proteomes of these bacteria, which led us to identify a putative endopeptidase called SagA (Fig. 4). We heterologously produced SagA and confirmed that the enzyme can degrade peptidoglycan and inhibit M. catarrhalis growth *in vitro* (Fig. 5). Finally, we demonstrated that *Rothia* can reduce M. catarrhalis levels in an airway epithelial cell model (Fig. 6).

*Rothia* species, including two of the three highlighted in our study, are generally considered to be inhabitants of the oral cavity (58). In a recent survey, isolates of *R. dentocariosa* and *R. mucilaginosa* were commonly cultured from pharynx samples of healthy children. However, *R. mucilaginosa* was rarely cultured from nasopharynx samples and *R. dentocariosa* was not detected at this site (59). However, we were able to culture and identify *Rothia* isolates from approximately half of the nasal lavage specimens that we tested. In addition, *Rothia* are regularly identified by sequencing studies as low-abundance members of upper and lower respiratory tract microbiomes. The relative abundances we observed were similar to what has been reported for other 16S rRNA gene amplicon sequencing studies of the anterior nares, nasal cavity, and nasopharynx (60–62). Together, these findings support the conclusion that *Rothia* are indeed bona fide inhabitants of the human nasal microbiome.

Prior research on *Rothia* has largely focused on their potential as opportunistic pathogens, primarily in immunocompromised hosts (63). However, *Rothia* have more recently been viewed as commensal bacteria and perhaps even mutualists in the oral and respiratory tracts. For instance, colonization of either an alveolar epithelial cell model or mouse lung with *R. mucilaginosa* prevented a P. aeruginosa-mediated, lipopolysaccharide-induced inflammatory response, resulting in reduced tissue damage compared to controls (64). Within induced sputum from individuals with bronchiectasis, the absolute abundance of *R. mucilaginosa* was inversely correlated with the levels of the proinflammatory markers interleukin 1 beta and interleukin 8 (64). The enzymatic activity of SagA itself may also trigger beneficial immune signaling in hosts. In a subcutaneous melanoma model, mice whose gastrointestinal tracts had been colonized by SagA-producing enterococci had significantly reduced tumor volume when later administered the immune checkpoint inhibitor anti-PD-L1, compared to mice treated with anti-PD-L1 alone. This antitumor effect was shown to be dependent on activation of the innate immune sensor nucleotide-binding oligomerization domain-2 (NOD2). The NOD2 sensor is activated by *N*-acetylglucosamine muramyl dipeptides generated from peptidoglycan by the d,l-endopeptidase activity of enterococcal SagA, suggesting that the microbiota can modulate responses to immunotherapy (65). In addition, NOD2 plays a role in the immune response to otitis media (66), the pulmonary immune response (67), and pathogen clearance (68). These examples, together with our findings, suggest a new role for *Rothia* as mutualists that defend against the pathobiont M. catarrhalis and modulate the host immune response. Subsequent work will be required to validate the role *Rothia* spp. play in these processes *in vivo*.

We do not fully understand how SagA acts on M. catarrhalis. In general, the outer membrane of Gram-negative bacteria, including M. catarrhalis, excludes molecules as large as SagA (~17.2 kDa). However, human lysozyme C (~14.7 kDa) has been previously shown to be active against M. catarrhalis (69). Lysozyme targets the peptidoglycan, catalyzing hydrolysis of the 1,4-beta linkages between *N*-acetylmuramic acid and *N*-acetylglucosamine residues, whereas SagA presumably uses its endopeptidase activity to target the pentapeptide stem between layers of peptidoglycan. The precise mechanism used by lysozyme to overcome the outer membrane is not fully known, but potentially involves membrane disruption through its innate cationic property at the nasal mucosal pH of 6.5 (calculated charge [*Z*] = +7.8) (70). However, unlike lysozyme, the secreted fragment of SagA is anionic at pH 6.5 (calculated *Z* = –3.2). Perhaps *Rothia* produce their own cationic peptides that act to permeabilize the outer membrane to SagA, or take advantage of a host-produced protein *in vivo*. In the latter case, one such possibility is lactoferrin, which is known to permeabilize the outer membrane of Gram-negative bacteria (71) and is present at high concentrations within nasal secretions (72). Therefore, further work is required to determine how SagA gains access to the peptidoglycan of M. catarrhalis. Moreover, additional work is necessary to determine why *Moraxella* species were more susceptible to inhibition by *Rothia* than any of the other bacteria that we tested, including other closely related *Gammaproteobacteria*.

Here, we note an inconsistency between our *in vitro* and ALI cell culture results. We found that *R. aeria* tended to be non-inhibitory toward M. catarrhalis *in vitro*. However, *R. aeria* strain RSM41, which we chose as a representative of this species because it almost never inhibited M. catarrhalis in inhibition assays, was equally protective against M. catarrhalis as *R. similmucilaginosa* in ALI cell cultures. Although *R. aeria* tended to secrete less SagA than *R. dentocariosa* and *R. mucilaginosa*, it is possible that *R. aeria* produces sufficient quantities of the enzyme when cultured on epithelial cells, the anti-M. catarrhalis activity of SagA is potentiated by a host-produced factor (see above), or *Rothia* produces additional proteins that are responsible for M. catarrhalis inhibition on cells.

In conclusion, we report that *Rothia* species potentially inhibit colonization of the nasal cavity by M. catarrhalis using an interference competition mechanism by producing a secreted peptidoglycan endopeptidase that directly inhibits M. catarrhalis growth. Given the specificity of these *Rothia* against *Moraxella* spp., we suggest that these bacteria or SagA itself could be developed as therapeutics to selectively inhibit nasal colonization by M. catarrhalis. Furthermore, we suspect that additional efforts to screen the nasal microbiota for bacteria that produce antimicrobials will yield more examples of proteins or metabolites with anti-M. catarrhalis activity.

## MATERIALS AND METHODS

### Nasal specimen collection and cold severity scoring.

The nasal lavage specimens used in this study were collected from children and banked as part of the RhinoGen study (25, 43). Informed consent was obtained from guardians, and the Human Subjects Committee at the University of Wisconsin-Madison approved the study (institutional review board [IRB] approval no. H-2007-0136-CR008). The cold severity score was determined after sample collection. This score is the summation of daily symptom scores for the period of the illness. Specimens collected within ±3 days of illness symptoms were considered to be associated with that illness. The daily scores ranged from 0 for absent to 3 for severe respiratory symptoms (25) (Table S1).

### DNA extraction and 16S rRNA gene V4 region amplicon sequencing.

We extracted total DNA from nasal lavage samples using the Bacteremia DNA isolation kit (MO BIO Laboratories Inc.). We included a negative extraction control of sterile water treated identically to the samples. All samples were quantified using a Qubit fluorometer with broad range standards (Invitrogen). Initially, we attempted to directly amplify the 16S rRNA gene V4 region from the extracted total DNA. However, we did not observe amplification after electrophoresis of PCR products on a 1% (wt/vol) agarose Tris-acetate-EDTA (TAE) gel. Therefore, we decided to use a nested PCR approach.

We first amplified the near full-length 16S *rRNA* gene in triplicate from the extracted samples with primers 27F (5′-AGAGTTTGATCCTGGCTCAG-3′) and 1492R (5′-GGTTACCTTGTTACGACTT-3′) using KAPA HiFi HotStart DNA polymerase (Roche). Each 25-μL reaction mixture contained 5 μL of extracted DNA (median: 34 ng, IQR: 3.8 to 110 ng) and 20 pmol of each primer. We used the following PCR cycling conditions: 95°C for 1 min; 15 cycles of 95°C for 30 s, 58°C for 30 s, and 72°C for 30 s; and a final extension at 72°C for 5 min. Subsequently, we amplified the 16S *rRNA* gene V4 region in triplicate using the PCR products as the template with primers 515F (5′-[P5 Illumina Adapter]-[Index]-TATGGTAATTGTGTGCCAGCMGCCGCGGTAA-3′) and 806R (5′-[P7 Illumina Adapter]-[Index]-AGTCAGTCAGCCGGACTACHVGGGTWTCTAAT-3′). Each 25-μL reaction mixture contained 2 μL of the unpurified first-round PCR product and 10 pmol of each primer. We used the same PCR cycling conditions as above. We electrophoresed 45 μL of pooled triplicated second-round PCR products on a 1.2% (wt/vol) agarose 0.5× Tris-borate-EDTA (TBE) gel at 100 V for 1 h. Next, we purified the PCR product from the agarose gel using the Wizard SV Gel and PCR Clean-Up System (Promega). We quantified DNA as described above. In the negative extraction control, the DNA concentration was below the limit of detection. We pooled 2 ng of each sample together with 1 μL of the undiluted negative extraction control. The amplicon libraries were prepared and sequenced using the 2× 300-bp paired-end Illumina MiSeq platform at the University of Wisconsin-Madison Biotechnology Center (Madison, WI).

### Bacterial community analysis.

We used fastp version 0.20.0 (73) for quality control and preprocessing of the raw amplicon sequencing reads. We used the mothur version 1.44.1 pipeline (74) for read processing, assembly, alignment, and classification. For sequence classification, we used the expanded human oral microbiome database 16S *rRNA* RefSeq version 15.1 (3). We defined OTUs using a 97% sequence similarity threshold (Table S2). There was >99% coverage of these samples, as assessed by Good’s Coverage Metric (Table S1). For all subsequent analyses, we used R version 4.0.4 (75). We processed the OTU table generated by mothur and subtracted the reads from each OTU in each sample corresponding to the number of reads detected in the negative extraction control. If OTU counts became negative after subtraction, we replaced the negative value with 0. We removed OTUs represented by <10 total reads from the data set. We then converted OTU counts to relative abundances and used the phyloseq package version 1.33.0 (76) and the vegan package version 2.5–6 (77) in R to perform NMDS and ANOSIM based on the Bray-Curtis dissimilarity between nasal bacterial communities. We used the envfit function from vegan to fit OTU vectors onto the ordination plot.

### Bacterial isolation, culturing, and initial identification.

To obtain isolates for culture-based work, we diluted 200 μL of thawed nasal lavage specimens with 800 μL of sterile phosphate-buffered saline (PBS) and then inoculated 100 μL onto 25-mL BHI (DOT Scientific) plates (diameter: 8.6 cm) solidified with 1.5% [wt/vol] agar (VWR). In addition to using standard BHI plates, we also included plates with 50 μg/mL lithium mupirocin (Sigma-Aldrich) to inhibit the growth of Staphylococcus spp. and allow for isolation of slower-growing organisms and 25 μg/mL vancomycin (Sigma-Aldrich) to inhibit the growth of Gram-positive bacteria and enrich for M. catarrhalis. Each sample was plated in triplicate onto both medium types. We incubated plates aerobically at 37°C for 1 week. We selected ≥2 colonies of each distinct morphotype per plate and passaged the isolates aerobically on BHI plates with no antibiotic supplementation at 37°C until we obtained pure cultures. All bacterial isolates were cryopreserved at −80°C in 25% (vol/vol) glycerol. Bacterial strains used in this study are listed in Table S3 in the supplemental material. For routine culturing and all following assays, we used BBL BHI (Becton, Dickinson and Co. [BD]) solidified with 1.5% (wt/vol) Bacto agar (BD) for plates because we observed poor or inconsistent growth of M. catarrhalis on BHI or agar produced by other manufacturers. For experiments testing the effect of iron on inhibition, we supplemented the BHI agar plates with 200 μM FeCl_3_ (Fisher Scientific).

10.1128/mbio.00464-23.3 TABLE S3 Bacterial strains used in this study. Download Table S3, DOCX file, 0.01 MB. Copyright © 2023 Stubbendieck et al. 2023 Stubbendieck et al. https://creativecommons.org/licenses/by/4.0/ This content is distributed under the terms of the Creative Commons Attribution 4.0 International license.

To identify isolates to the genus level, we performed colony PCR using the universal 27F (5′-AGAGTTTGATCMTGGCTCAG-3′) and 1,492R (5′-CGGTTACCTTGTTACGACTT-3′) primers (78). Briefly, we suspended a small portion of each bacterial colony or liquid culture in 20 μL of 0.02 N sodium hydroxide and incubated the samples at 95°C for 10 min. We then chilled the samples on ice and centrifuged them at 21,130 × *g* for 5 min and used 1 μL of the supernatant as the template for PCR. We sequenced the PCR products using the Sanger method at the University of Wisconsin Biotechnology Center DNA Sequencing Facility (Madison, WI) and identified isolates to the genus level with the Ribosomal Database Project Classifier (79).

### Inhibition assays.

To determine whether *Rothia* isolates inhibited M. catarrhalis and other *Gammaproteobacteria*, we used coculture plate inhibition assays. We inoculated 3 mL BHI broth with single colonies of *Rothia* from 2-day-old plates and incubated the cultures at 37°C while shaking. After overnight growth, we diluted each culture to an optical density at 600 nm (OD_600_) of 0.05 in 3 mL fresh BHI, incubated the cultures until they reached an OD_600_ of 0.4 to 0.6, and spread 50 μL in a ~6 × ~2 cm-wide line in the center of a 25-mL BHI agar plate (diameter: 8.6 cm). We incubated the plates aerobically for 3 days at 37°C. We prepared overnight cultures of M. catarrhalis and other *Gammaproteobacteria* by inoculating single colonies into 3 mL BHI broth and incubated the cultures at 37°C with shaking. After overnight growth, we diluted the cultures to an OD_600_ of 0.5 in fresh BHI and spotted 5 μL approximately 0.5 cm away from the line of *Rothia*. When the spots dried, we returned the plates to 37°C, then scored them for inhibition after overnight incubation. All inhibition assays were experimentally replicated ≥3 times, with at least 2 technical replicates per experiment.

### Genomic DNA extraction and sequencing.

We used either the DNA Miniprep kit (ZymoBIOMICS) with 2× 5-min bead beating steps or the MasterPure Yeast DNA purification kit (Lucigen) with the addition of 1 μL Ready-Lyse Lysozyme Solution (Lucigen) and 1 μL 5 mg/mL RNase A (Lucigen) to lyse *Rothia* isolates and purify genomic DNA. Libraries were prepared with a single library preparation method based on the Illumina Nextera kit and sequenced using the 2× 150-bp paired-end Illumina NextSeq 2000 platform at SeqCenter (Pittsburgh, PA).

### Genome assembly and annotation.

We processed raw genomic reads using fastp 0.20.0 and assembled draft genome sequences using SPAdes 3.11.1 (80), using default parameters for both. To identify homologues present across *Rothia* genomes, we downloaded high-quality complete genome sequences of *R. aeria*, *R. dentocariosa*, *R. kristinae*, *R. mucilaginosa*, *R. nasimurium*, and *R. terrae* from GenBank. We identified protein-coding genes with Prokka (81) using default parameters and used BuildGroups.py from PyParanoid (82) to build groups of homologous gene families. To predict secreted proteins, we submitted the consensus sequences for each homologous family identified by PyParanoid to the SignalP-5.0 webserver (https://services.healthtech.dtu.dk/service.php?SignalP-5.0) (83).

### Pangenome analysis.

To generate a pangenome, we used the anvi’o 7 pangenome analysis pipeline (84) with the following parameters: --minbit 0.5 --mcl-inflation 10 --use-ncbi-blast.

### Core-genome phylogeny.

We constructed a core-genome phylogeny of nasal *Rothia* isolates using core_species_tree.pl (https://github.com/chevrm/core_species_tree) as previously described (85). We used FigTree v1.4.3 (https://github.com/rambaut/figtree/) to root the phylogeny on Kocuria rosea ATCC 186 and display the branch length based on proportional length to the root.

### Detection and identification of *Rothia* BGCs.

We identified BGCs in *Rothia* genomes using antiSMASH version 4.2.0 (50) with the following parameters: --clusterblast --knownclusterblast --smcogs. To identify relationships between the *Rothia* BGCs, we used BiG-SCAPE 1.0.1 (86) with the following parameters: --cutoffs 0.3 --include_singletons. We generated a presence-absence matrix of BGCs in *Rothia* using the clustering families identified by BiG-SCAPE.

### CAS assay.

We used the overlay CAS assay to test for siderophore production as previously described (87). We cultured *Rothia* for 3 days at 37°C, as above. We then overlaid each plate with 5 mL of CAS reagent overlay (52.5 μM CAS, 50 μM FeCl_3_, 0.5 mM hexadecyltrimethylammonium bromide, 10 mM piperazine-N,N′-bis[2-ethanesulfonic acid], 1% [wt/vol] agarose [pH 6.8]) (88) and incubated the plates at ambient temperature in the dark for 4 h before scanning them for color change, which would indicate the production of a siderophore (89).

### Extraction of secreted proteins.

To identify proteins secreted by *Rothia* isolates into agar, we adapted a previously used protocol (90). We cultured *Rothia* onto six 25-mL BHI plates, as above. After incubation for 3 days at 37°C, we used clean razor blades to excise the agar from a distance of ~0.1 to ~1 cm away from both sides of the *Rothia* without disturbing the bacterial colony.

We centrifuged the agar slices at 4,000 × *g* for 10 min at ambient temperature, then combined them with an equal volume of extraction buffer (50 mM Tris-HCl, 0.2% [wt/vol] *N*-lauroylsarcosine sodium salt [pH 7.5]) and incubated the mixture at ambient temperature on a shaking platform set at 250 rpm overnight. We then centrifuged the samples at 4,000 × *g* for 20 min at ambient temperature and filtered the supernatant through cheesecloth to remove agar. We added trichloroacetic acid (TCA) to a final concentration of 7.5% (wt/vol) and precipitated protein overnight at −20°C. We pelleted the precipitated protein by centrifuging the samples at 4,000 × *g* for 30 min at 4°C. We then washed the protein pellet three times with 1 mL acetone and centrifugation at 21,130 × *g* for 5 min at 4°C. We evaporated residual acetone under a laminar flow hood. To remove residual agar from the samples, we first resuspended the protein pellets in 250 μL of 50 mM Tris-HCl (pH 7.5) and centrifuged the samples at 21,130 × *g* for 30 min at 4°C. We then transferred the supernatant into a new tube, and centrifuged the samples overnight at 21,130 × *g* at 4°C. Finally, the supernatant was transferred into a new tube and protein content was measured using a Coomassie protein assay (Thermo Fisher Scientific). We analyzed 3 μg of each protein sample by SDS-PAGE using a 12% Criterion TGX precast midi protein gel. We visualized proteins by staining with RAPIDstrain (G-Biosciences) for 1 h followed by 3× 15-min destaining washes in distilled water.

### Identification of secreted proteins.

We submitted protein samples for digestion and LC-MS/MS analysis at the University of Wisconsin Biotechnology Center Mass Spectrometry Facility (Madison, WI). To precipitate proteins, 60 μL of each sample was treated with a mixture of 10% TCA/50% acetone/40% water (vol/vol/vol) for 30 min on ice. The samples were centrifuged at 16,000 × *g* for 10 min and the pellet was washed with 800 μL acetone at −20°C. The samples were then centrifuged as described above and washed with 800 μL acetone at −20°C. Subsequently, the pellets were resolubilized and denatured in 50 μL of a solution containing 8 M urea, 50 mM ammonium bicarbonate (NH_4_HCO_3_), and 1 mM Tris-HCl (pH 8.5), then sonicated for 1 min. The proteins were quantified from 10 μL of the sample with the Pierce 660 nm Protein Assay reagent. The remainder of the sample was reduced by combining it with 5 μL of 25 mM dithiothreitol (DTT), 10 μL of methanol, and 65 μL of NH_4_HCO_3_ (pH 8.5). The samples were incubated at 52°C for 15 min, then cooled on ice before being alkylated with 6 μL of 55 mM chloroacetamide for 15 min in the darkness at ambient temperature. The alkylation reaction was quenched with 16 μL of 25 mM DTT. To digest the proteins, 14 μL of a trypsin/LysC solution (100 ng/μL of 1:1 trypsin [Promega] and LysC [FujiFilm] mix in 25 mM NH_4_HCO_3_) and 44 μL of 25 mM NH_4_HCO_3_ (pH 8.5) was added to the samples and incubated for 2 h at 42°C. Afterwards, 7 μL of the trypsin/LysC solution was added to the samples and digestion continued at 37°C overnight. The digestion reaction was terminated by addition of trifluoroacetic acid (TFA) to a final concentration of 0.3% [vol/vol].

The digested and acidified samples were cleaned using OMIX C18 SPE cartridges (Agilent) following the manufacturer’s protocol, eluted with 20 μL of 50% (vol/vol) acetonitrile (ACN) with 0.1% (vol/vol) TFA, dried, and reconstituted in 40 μL 2% (vol/vol) ACN with 0.1% (vol/vol) formic acid. Peptides were analyzed by nanoLC-MS/MS using the Agilent 1100 nanoflow system (Agilent) connected to an LTQ-Orbitrap Elite (Thermo Fisher Scientific) equipped with an EASY-Spray electrospray source (held at constant 35°C). Chromatography of peptides prior to mass spectral analysis was accomplished using a capillary emitter column (PepMap C18, 3 μM, 100 Å, 150 × 0.075 mm, Thermo Fisher Scientific) onto which 2 μL of the extracted peptides was automatically loaded. A NanoHPLC (high-performance liquid chromatography) system delivered 0.1% (vol/vol) formic acid (Solvent A), and 99.9% (vol/vol) ACN with 0.1% (vol/vol) formic acid (Solvent B) at a flow rate of 0.50 μL/min to load the peptides over a 30-min period. The flow rate was adjusted to 0.3 μL/min to elute peptides directly into the nano-electrospray with a gradual gradient of 0% to 30% B over 78 min and concluded with a 5-min fast gradient from 30% to 50% Bm at which time a 5-min flash-out from 50% to 95% B took place. As peptides eluted from the HPLC column/electrospray source survey, MS scans were acquired in the Orbitrap at a resolution of 120,000, followed by CID-type MS/MS fragmentation of the 30 most intense peptides detected in the MS scan from 350 to 1,500 *m/z*. Redundancy was limited by dynamic exclusion.

The raw MS/MS data files were converted to the mgf file format using MSConvert (ProteoWizard) and searched against user-defined databases for each *Rothia* strain along with a cRAP common lab contaminant database using in-house Mascot search engine version 2.7.0 (Matrix Science) with variable methionine oxidation plus asparagine or glutamine deamidation and fixed cysteine carbamidomethylation. The peptide mass tolerance was set at 10 ppm and fragment mass at 0.6 Da. Protein annotations, significance of identification, and spectral-based quantification was determined with Scaffold version 4.11.0 (Proteome Software Inc.). The protein identifications were accepted if they could achieve a false discovery rate of <1.0% and contained at least 3 identified peptides. Protein probabilities were assigned by the Protein Prophet algorithm (91). Proteins that contained similar peptides and could not be differentiated based on MS/MS alone were grouped to satisfy the principles of parsimony. Proteins which shared significant peptide evidence were grouped into clusters.

### Comparison of secreted proteomes.

We mapped the spectral counts for each protein identified by LC-MS/MS onto the homolog groups generated using PyParanoid described above. In cases where we identified multiple proteins within the same homolog group from the same strain, we aggregated the spectral counts into a single count for that homolog group. We transformed spectral counts into a relative abundance by dividing each count by the total number of spectral counts identified in a sample. We then filtered out groups of potentially contaminating cytoplasmic proteins based on the presence of a signal peptide as determined by SignalP. We used the phyloseq and vegan packages in R to perform NMDS and ANOSIM and to fit homolog group vectors onto the ordination plot as above.

### SagA sequence alignment.

We aligned the primary sequences of SagA from *R. aeria* strain RSM41, *R. dentocariosa* strain RSM522, and *R. similmucilaginosa* strain RSM42 against structurally characterized C40 family peptidase homologues using MEGA X (92). We visualized the alignment using the ggmsa package (93) in R.

### SagA structure modeling.

We used the Phyre2 webserver (http://www.sbg.bio.ic.ac.uk/phyre2/) (94) to generate a structure of SagA by modeling 176 amino acids (89% of the primary sequence) from *R. similmucilaginosa* onto the hypothetical invasion protein of M. tuberculosis (PDB ID: 2XIV). We visualized this structural model using PyMol version 2.5.0 (95).

### Generating *sagA* constructs.

For initial experiments, we cloned *sagA* from *R. similmucilaginosa* strain RSM42 using primers oRSC91 (5′-AGGAGGAATTAACTATGGATCTGTCCTACGACTACACC-3′) and oRSC92 (5′-CCGCCAAAACAGCCAAGCTTTTAGAGAGCGGTGTAGTAGC-3′) and the vector pBAD30 using primers oRSC93 (5′-AAGCTTGGCTGTTTTGGCGG-3′) and oRSC94 (5′-CATAGTTAATTCCTCCTGAATTCGCTAGCCCAAAAAAACG-3′). These primers contained overhangs for assembly and included an optimal E. coli Shine Dalgarno sequence. We combined these fragments together using Gibson assembly (96) and transformed the products into the chemically competent E. coli strain NEB 5α to generate strain RSC0026. For subsequent experiments, we ordered a codon-optimized *sagA* sequence with an in-frame coding sequence for an N-terminal 6×His tag as a single gene block (GenScript). We amplified this gene block using primers oRSC130 (5′-ATGCGAATTCAGGAGGTCATCATGCACCATCATCATCACCATGA-3′) and oRSC131 (5′-ATGCAAGCTTTCAGAGGGCGGTGTAATACC-3′). These primers contained overhangs with an optimal E. coli Shine Dalgarno sequence and restriction sites for EcoRI and HindIII, respectively. We cloned the digested gene block into pBAD30 using EcoRI and HindIII (New England Biolabs) and transformed the product into the chemically competent E. coli strain Top10 to generate strain RSC0060. All plasmids were validated using whole plasmid sequencing at plasmidsaurus (Eugene, OR).

### Heterologous production and purification of SagA^37-197^.

We inoculated 250 mL of terrific broth (2.4% [wt/vol] yeast extract, 2% [wt/vol] tryptone, 0.4% [vol/vol] glycerol, 17 mM KH_2_PO_4_, 72 mM K_2_HPO_4_) with 250 μL of an overnight culture of E. coli strain RSC0060 and incubated the culture at 37°C while shaking at 250 rpm. When the cultures reached an OD_600_ of 0.1, we induced production of 6×His-SagA^37-197^ with a final concentration of 0.2% (wt/vol) l-arabinose, then continued incubation while shaking for 24 h. Subsequently, we collected the cells by centrifugation at 4,000 × *g* at 4°C for 30 min. We partially purified 6×His-SagA^37-197^ from the cell pellet using a Ni-NTA Fast Start kit (Qiagen) following the manufacturer’s protocol under native conditions, with the addition of lysis by sonication.

### Zymography assay.

To prepare the substrate for zymography, we inoculated 500 mL BHI with 1 mL of a M. catarrhalis overnight culture and incubated the cultures at 37°C while shaking. When the cultures reached an OD_600_ of 1.0, we collected the cells by centrifugation at 4,000 × *g* at 4°C for 30 min. We resuspended the cell pellets in 50 mL wash buffer (20 mM Tris-HCl, 100 mM NaCl [pH 7.5]). We then centrifuged the cell suspensions as described above, resuspended them in 10 mL of 1.5 M Tris-HCl (pH 8.8), and stored 1-mL aliquots at −20°C until use. We prepared gels with standard protocols using the Laemmli buffer system, with gels containing 20% (wt/vol) polyacrylamide in the resolving gel and 4% (wt/vol) polyacrylamide in the stacking gel. For zymography gels, during polymerization we incorporated M. catarrhalis cells which had been previously boiled for 10 min into the polyacrylamide gel at a final concentration of 10% (vol/vol).

We boiled 7.5 μL of protein samples with 2.5 μL 4× loading buffer (200 mM Tris-HCl, 8% [wt/vol] SDS, 40% [vol/vol] glycerol, trace bromophenol blue) for 10 min. We then electrophoresed these samples at 100 V for 4 h. All subsequent steps occurred with gentle rocking at ambient temperature unless otherwise noted. We washed the zymogram gel twice in 100 mL double-distilled water (ddH_2_O) for 30 min. We then incubated the gel in 100 mL of renaturation buffer (20 mM Tris, 50 mM NaCl, 20 mM MgCl_2_, 0.5% [vol/vol] Triton X-100 [pH 7.4]) for 1 h. After replacing the renaturation buffer with 100 mL fresh renaturation buffer, we incubated the zymogram for 12 h at 37°C. We stained the gel with 100 mL of staining solution (0.1% [wt/vol] methylene blue, 0.01% [wt/vol] KOH) for 1 h. We subsequently destained the gel with repeated washes in ddH_2_O. We scored peptidoglycan degradation as zones of clearing within the stained gel (97).

### Inhibition of *M. catarrhalis* using 6×His-SagA^37-197^.

We cultured M. catarrhalis strain O35E as above and diluted the culture to an OD_600_ of 0.01 in BHI. We then transferred 50 μL of culture into the wells of a 96-well plate containing either purified 6×His-SagA^37-197^ or an equivalent purification from an E. coli strain Top10 culture harboring an empty pBAD30 vector as a negative control. We incubated the plate at 37°C in a microplate spectrophotometer (Biotek) with continuous orbital shaking for 13 h and measured the OD_600_ of each well at 1-h intervals.

### ALI culture of airway epithelium.

Human bronchial and tracheal epithelial cells were obtained from residual tissue from lungs destined for transplantation in collaboration with the UW Health Lung Transplant Program. The protocol was reviewed by the University of Wisconsin IRB and was deemed “not human subjects research.” We thawed cryopreserved aliquots of cells and expanded them as monolayers in PneumaCult-Ex Plus Medium (StemCell Technologies) supplemented with 50 μg/mL gentamicin and 2 μg/mL fluconazole at 37°C in a 5% CO_2_ atmosphere. Once the cells reached 80% confluence, we transferred them to 12-well plates with Transwell semipermeable inserts (Corning) and allowed them to differentiate in PneumaCult-ALI medium (StemCell Technologies) supplemented with gentamicin and fluconazole, as above, at the ALI until ciliary motion was observed (≥21 days). We cultured the cells in an antibiotic-free medium 48 h prior to bacterial inoculation.

### Inhibition assays in airway epithelium cultures.

We centrifuged liquid cultures of *R. aeria* strain RSM41, *R. dentocariosa* strain RSM522, and *R. similmucilaginosa* strain RSM42 at 5,000 × *g* for 5 min and resuspended the cell pellet to an OD_600_ of ~0.25 in prewarmed PBS. We cultured M. catarrhalis on BD Chocolate Agar–GC II Agar with IsoVitaleX and prepared a suspension of the bacterial cells to an OD_600_ of ~0.25 in prewarmed PBS. We inoculated the apical surface of separate wells of ALI cultures with 50 μL of a *Rothia* suspension for 40 h. We infected ALI cells with M. catarrhalis as previously described (98). Briefly, we infected the apical surface with 50 μL of the M. catarrhalis suspension and incubated the cells at 37°C for 24 h. At the end of the infection period, we gently washed the apical surface with 500 μL prewarmed PBS and collected the cells in 350 μL of RLT Plus Buffer (Qiagen) containing 0.5% Reagent DX (Qiagen).

### Quantification of *M. catarrhalis* in airway epithelium cultures.

We transferred the cells to PowerBead tubes (Qiagen) for bead beating (5 min, 50 oscillations/s, Qiagen TissueLyser LT). We then extracted RNA and DNA as separate fractions using the AllPrep DNA/RNA Minikit (Qiagen), following the manufacturer’s instructions. We quantified M. catarrhalis *copB* DNA using real-time PCR on a 7500 Real Time PCR System (Applied Biosystems) using the specific primers *copB*-F (5′-GTGAGTGCCGCTTTACAACC-3′) and *copB*-R (5′-TGTATCGCCTGCCAAGACAA-3′), as previously described (99). We calibrated levels of *copB* to CFUe using a standard curve as previously described (98). Briefly, we cultured M. catarrhalis cultured overnight and quantified the CFU using standard methods. In parallel, we extracted DNA from an aliquot of the same culture using an AllPrep Power Viral kit (Qiagen) and prepared serial dilutions of the DNA corresponding to 10^2^ to 10^6^ CFU.

### Statistical analysis and data visualization.

All statistical analyses were performed in R with specific packages as described in the preceding sections. We generated graphics using ggplot2 (100) with some cleanup and assembly in InkScape.

### Data availability.

The raw amplicon sequences used for bacterial community analysis are deposited in the Short Read Archive under BioProject accession no. PRJNA866994. Genome sequences generated from this work are deposited under BioProject accession no. PRJNA867425. All other raw and derived data are available at https://doi.org/10.6084/m9.figshare.20444466. The scripts necessary to replicate this work are available at https://github.com/reedstubbendieck/rothia_moraxella.
