# Supplementary figures and images for: Rothia from the Human Nose Inhibit Moraxella catarrhalis Colonization with a Secreted Peptidoglycan Endopeptidase
Source: mBio. 2023 Apr 3;14(2):e00464-23. doi: 10.1128/mbio.00464-23 (PMC10128031; doi:10.1128/mbio.00464-23)

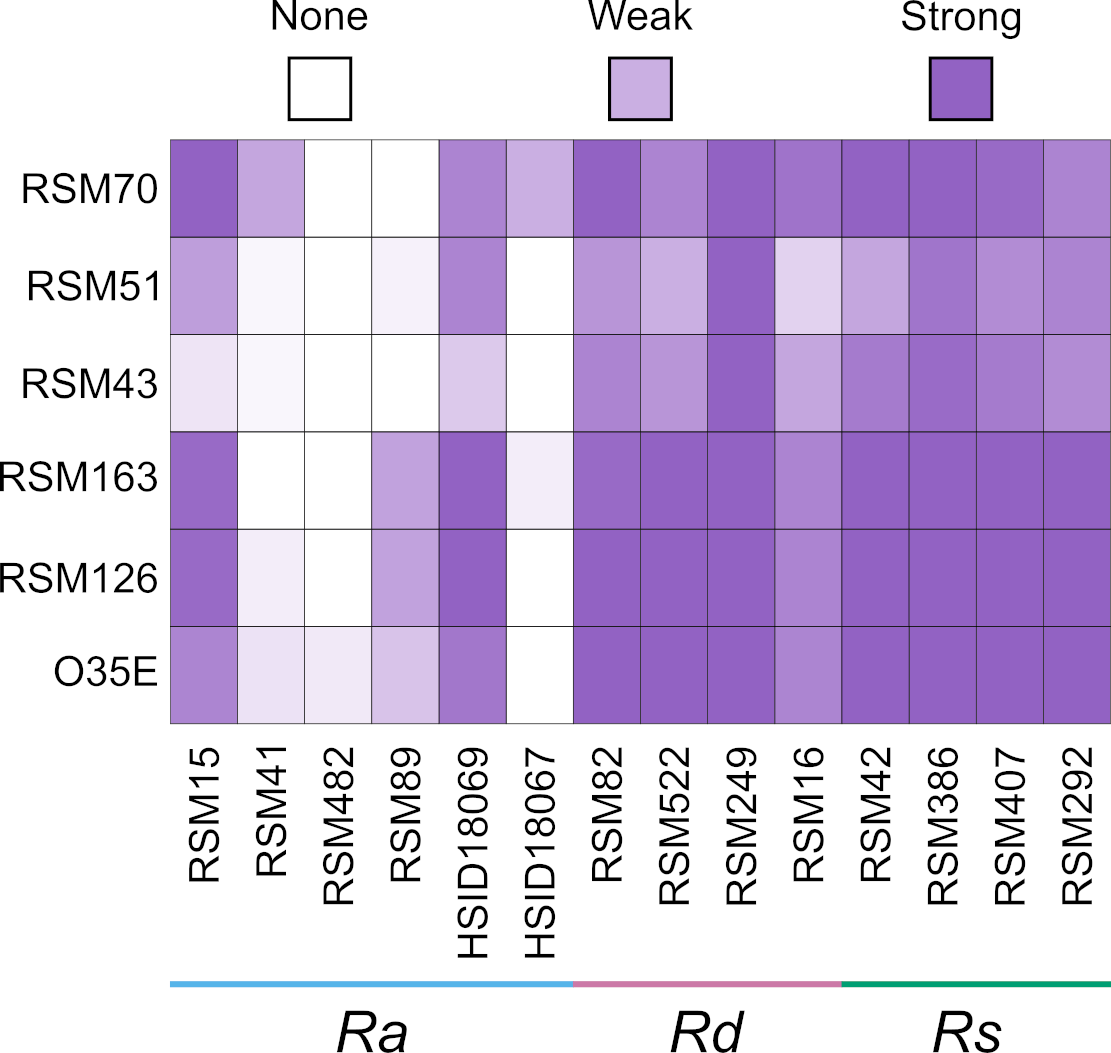

Supplement: FIG S1 [file mbio.00464-23-s0004.tif]

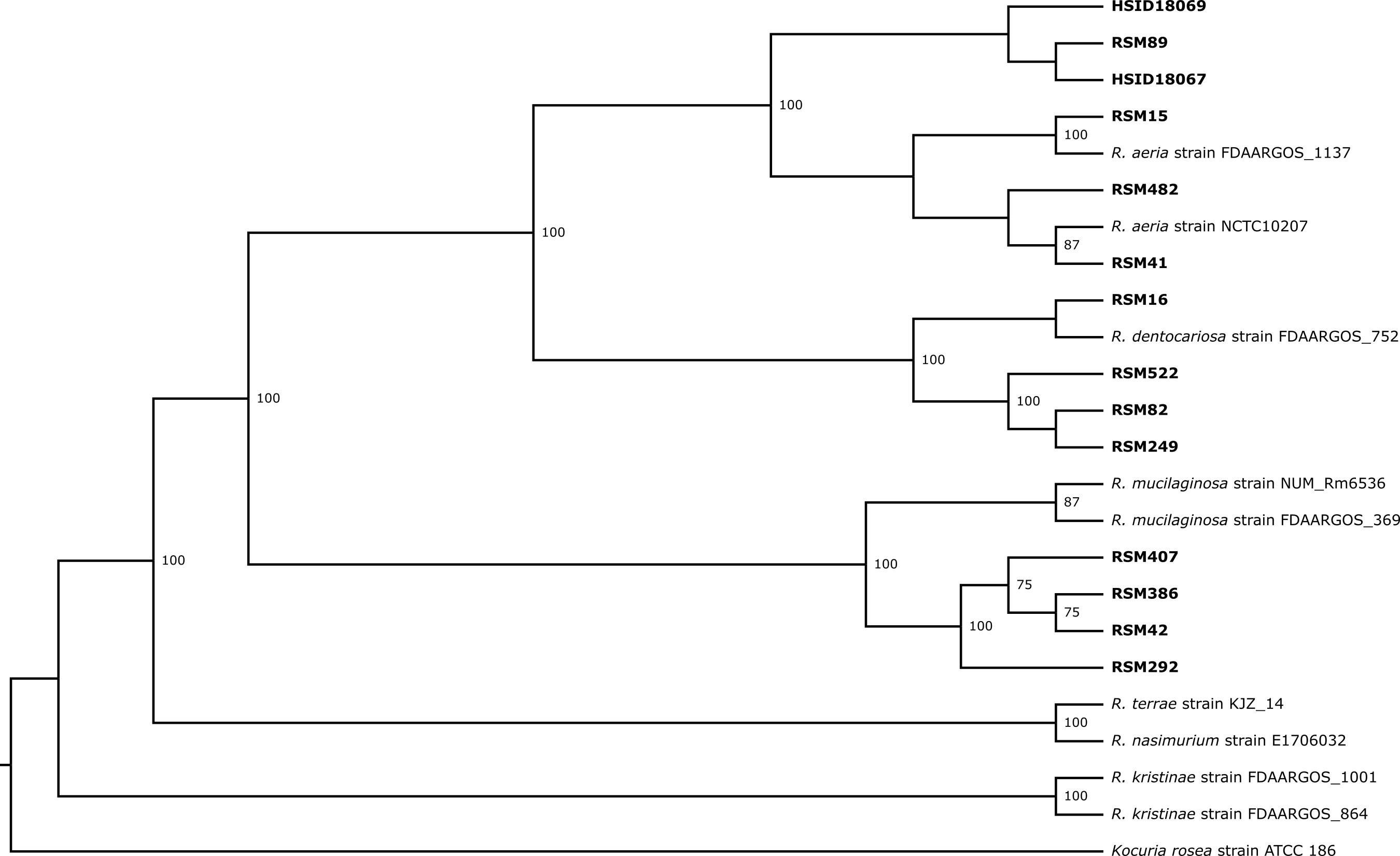

Supplement: FIG S2 [file mbio.00464-23-s0005.tif]

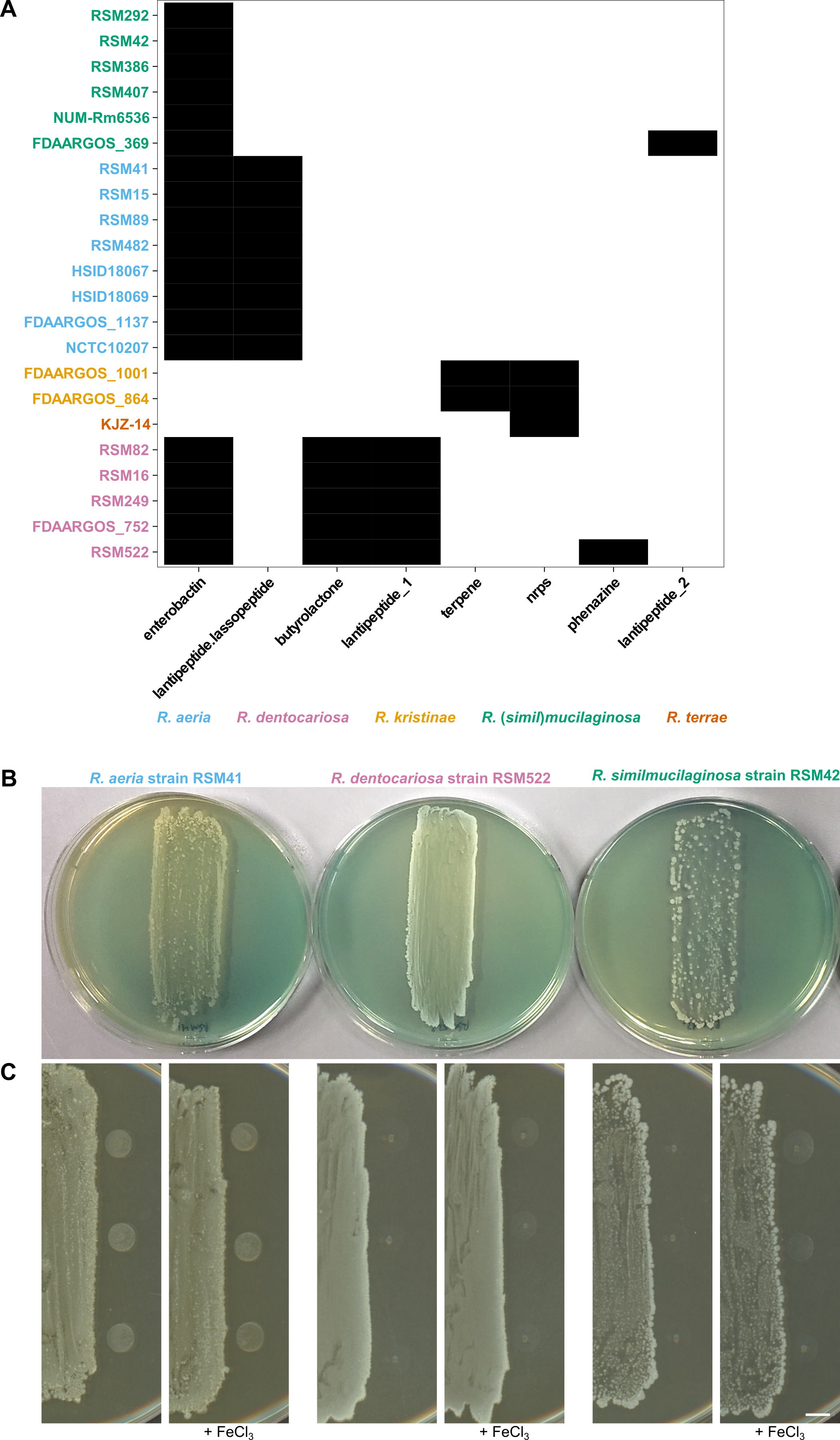

Supplement: FIG S3 [file mbio.00464-23-s0006.tif]

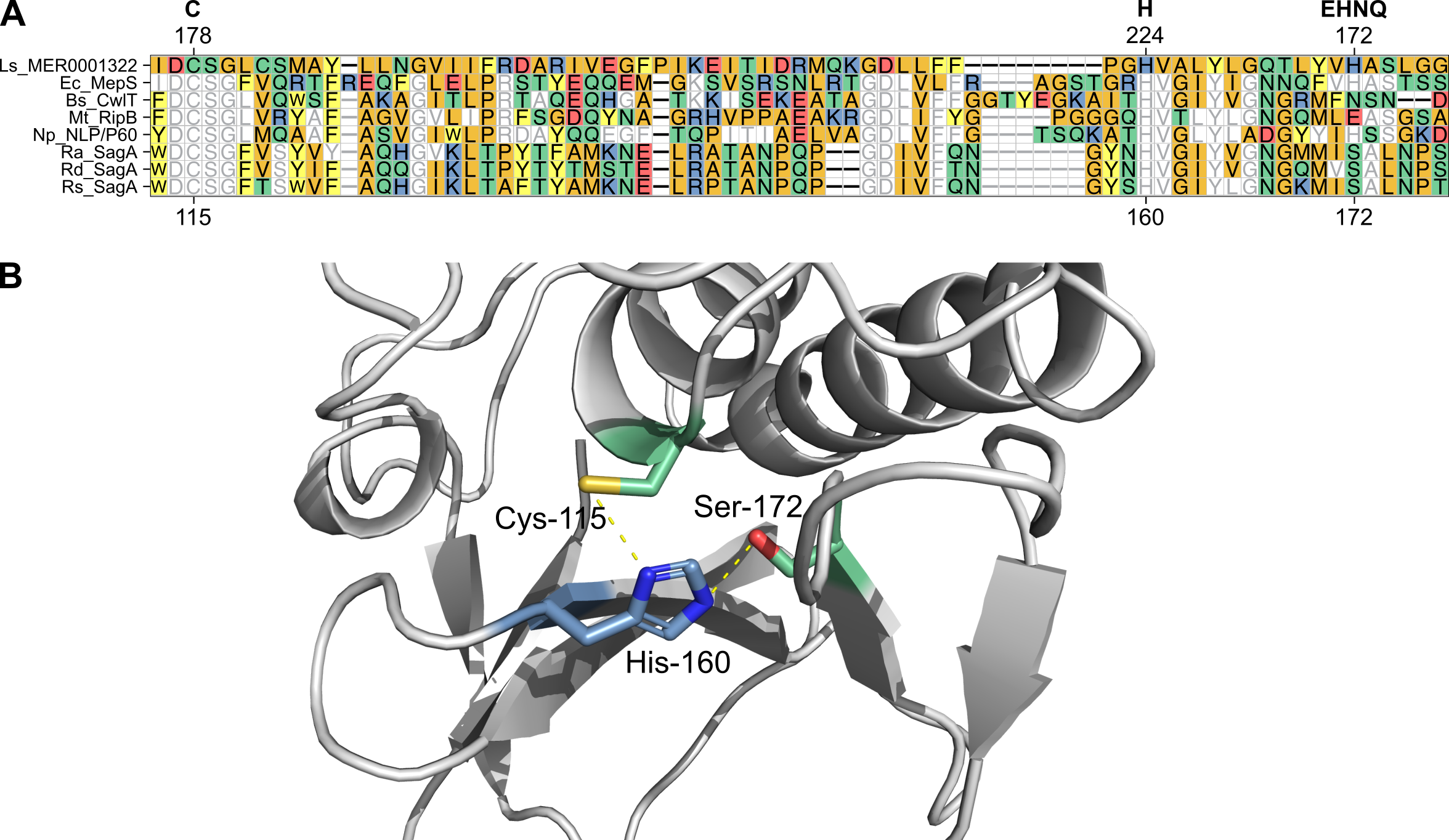

Supplement: FIG S4 [file mbio.00464-23-s0007.tif]

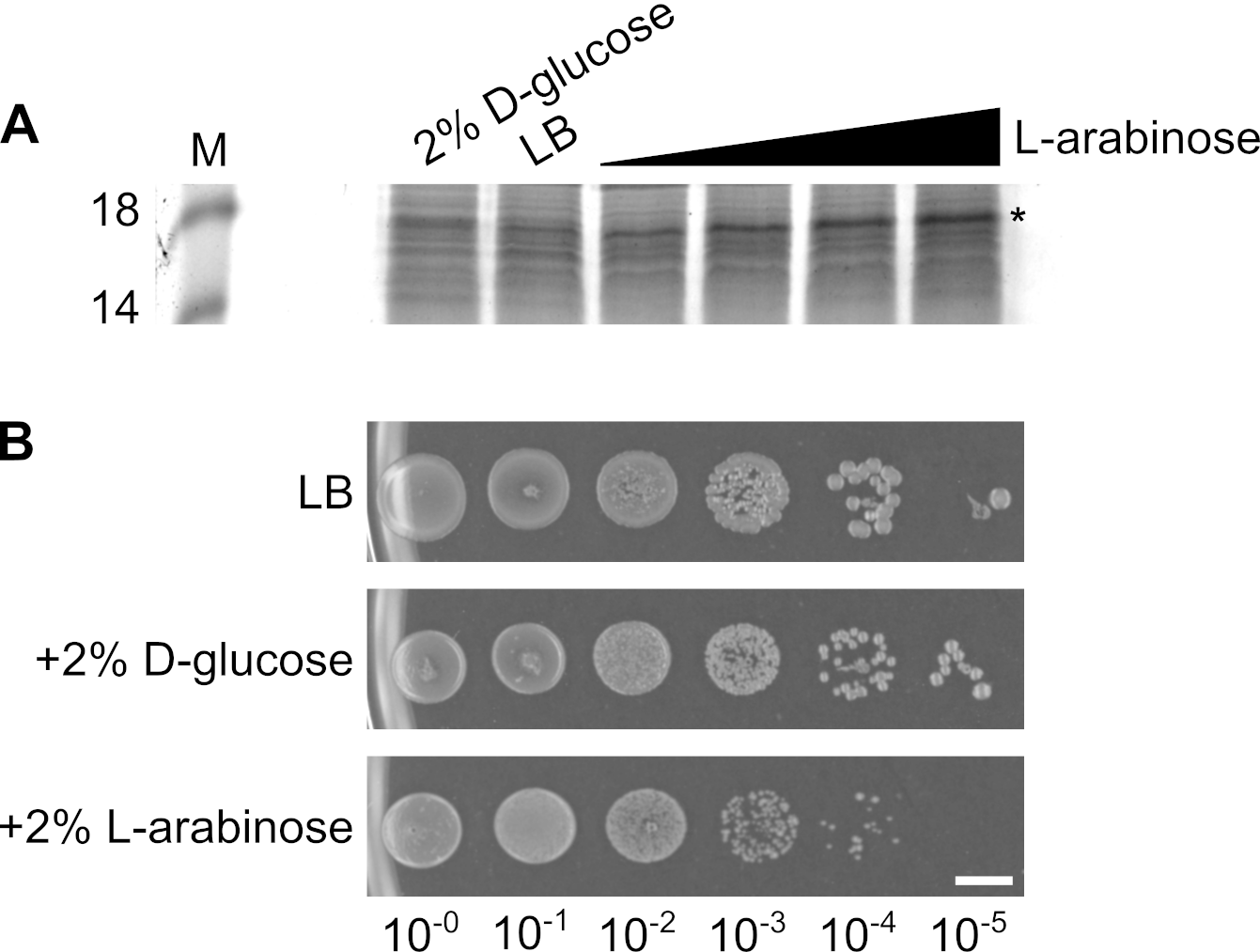

Supplement: FIG S5 [file mbio.00464-23-s0008.tif]
