# Supplementary material for: Rothia from the Human Nose Inhibit Moraxella catarrhalis Colonization with a Secreted Peptidoglycan Endopeptidase
Source: mBio. 2023 Apr 3;14(2):e00464-23. doi: 10.1128/mbio.00464-23 (PMC10128031; doi:10.1128/mbio.00464-23)
Supplement: TABLE S3 [file mbio.00464-23-s0003.docx]

**Table S3** Bacterial strains used in this study.

| **Strain** | **Organism** | **Reference/Source** |
| --- | --- | --- |
| ATCC BAA-1259 | *Moraxella bovoculi* | American Type Culture Collection |
| ATCC 19575 | *Moraxella ovis* | American Type Culture Collection |
| HSID18067 | *Rothia aeria* | Laboratory Collection/Reference 38 |
| HSID18069 | *Rothia aeria* | Laboratory Collection/Reference 38 |
| MG1665 | *Escherichia coli* | R. Welch (University of Wisconsin-Madison) |
| O35E^1^ | *Moraxella catarrhalis* | T. Murphy (State University of New York at Buffalo) |
| PID31 | *Serratia marcescens* | Laboratory Collection |
| PID38 | *Pseudomonas aeruginosa* PA01 | Laboratory Collection |
| PID42 | *Acinetobacter baumannii* | Laboratory Collection |
| PID45 | *Klebsiella pneumoniae* | Laboratory Collection |
| RSC0027 | *Escherichia coli* NEB 5α pBAD30Ω*sagA*(37-197) (*amp*) | This Study |
| RSC0060 | *Escherichia coli* Top10 pBAD30Ω6×His-*sagA*(37-197) (*amp*) | This Study |
| RSM15 | *Rothia aeria* | This Study |
| RSM16 | *Rothia dentocariosa* | This Study |
| RSM41 | *Rothia aeria* | This Study |
| RSM42 | *Rothia similmucilaginosa* | This Study |
| RSM43 | *Moraxella catarrhalis* | Laboratory Collection/Reference 98 |
| RSM51 | *Moraxella catarrhalis* | This Study |
| RSM70 | *Moraxella catarrhalis* | This Study |
| RSM82 | *Rothia dentocariosa* | This Study |
| RSM89 | *Rothia aeria* | This Study |
| RSM126 | *Moraxella catarrhalis* | This Study |
| RSM163 | *Moraxella catarrhalis* | Laboratory Collection/Reference 98 |
| RSM249 | *Rothia dentocariosa* | This Study |
| RSM292 | *Rothia similmucilaginosa* | This Study |
| RSM386 | *Rothia similmucilaginosa* | This Study |
| RSM407 | *Rothia similmucilaginosa* | This Study |
| RSM482 | *Rothia aeria* | This Study |
| RSM522 | *Rothia dentocariosa* | This Study |

^1^Also called 035E.
